# Dispersion, Rehybridization, and Pentacoordination: Keys to Understand Clustering of Boron and Aluminum Hydrides and Halides

**DOI:** 10.1021/acs.jpca.3c02747

**Published:** 2023-07-07

**Authors:** Otilia Mó, M. Merced Montero-Campillo, Manuel Yáñez, Ibon Alkorta, José Elguero

**Affiliations:** † Departamento de Química, Módulo 13, Facultad de Ciencias, and Institute of Advanced Chemical Sciences (IAdChem), Universidad Autónoma de Madrid, Campus de Excelencia UAM-CSIC, Cantoblanco, 28049 Madrid, Spain; ‡ Instituto de Química Médica, IQM-CSIC, Juan de la Cierva, 3, 28006 Madrid, Spain

## Abstract

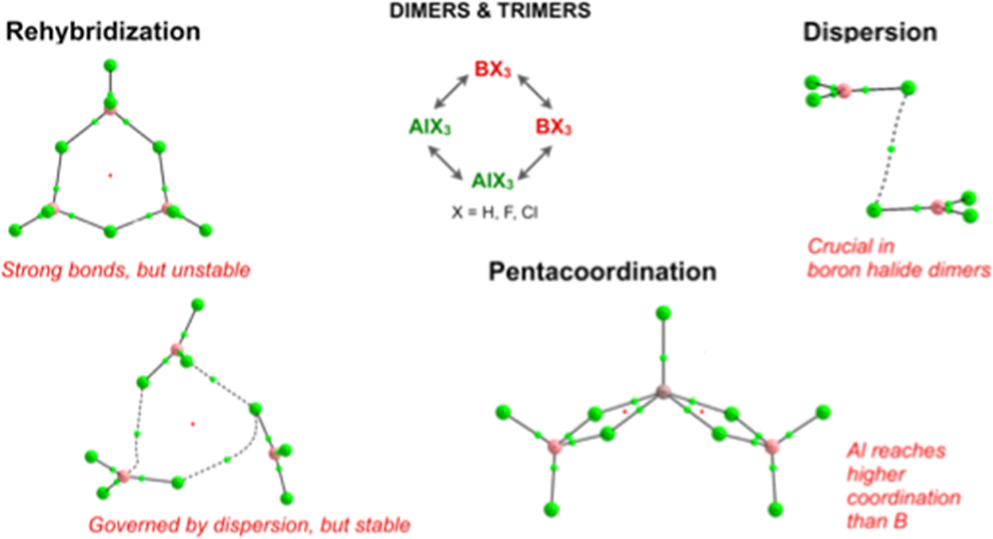

The structure, stability, and bonding characteristics of dimers and trimers involving BX_3_ and AlX_3_ (X = H, F, Cl) in the gas phase, many of them explored for the first time, were investigated using different DFT (B3LYP, B3LYP/D3BJ, and M06-2X) and ab initio (MP2 and G4) methods together with different energy decomposition formalisms, namely, many-body interaction-energy and localized molecular orbital energy decomposition analysis. The electron density of the clusters investigated was analyzed with QTAIM, electron localization function, NCIPLOT, and adaptive natural density partitioning approaches. Our results for triel hydride dimers and Al_2_X_6_ (X = F, Cl) clusters are in good agreement with previous studies in the literature, but in contrast with the general accepted idea that B_2_F_6_ and B_2_Cl_6_ do not exist, we have found that they are predicted to be weakly bound systems if dispersion interactions are conveniently accounted for in the theoretical schemes used. Dispersion interactions are also dominant in both homo- and heterotrimers involving boron halide monomers. Surprisingly, B_3_F_9_ and B_3_Cl_9_*C*_3*v*_ cyclic trimers, in spite of exhibiting rather strong B–X (X = F, Cl) interactions, were found to be unstable with respect to the isolated monomers due to the high energetic cost of the rehybridization of the B atom, which is larger than the two- and three-body stabilization contributions when the cyclic is formed. Another important feature is the enhanced stability of both homo- and heterotrimers in which Al is the central atom because Al is systematically pentacoordinated, whereas this is not the case when the central atom is B, which is only tri- or tetra-coordinated.

## Introduction

The role of quantum chemical modeling in understanding a great diversity of chemical or physical phenomena is nowadays an indisputable reality and reaches, as illustrated in a complete review by Raghavachari and Saha,^1^ very large systems such as proteins, clusters, and crystals. Throughout the past and present centuries, quantum chemical models have allowed to predict the existence of elusive molecules, determining their structures and describing the nature of their chemical bonds. A paradigmatic example of this predictive power is the Be_2_ molecule whose existence, as a weakly bound molecule, was predicted back in 1978^2^ and confirmed by means of full CI calculations^3^ in 1983. However, it was necessary to wait until the first years of the present century to have its first experimental characterization,^4^ its more accurate bonding characterization being reported by El Khatib et al. in 2014.^5^ Boron and aluminum are also elements that frequently present elusive compounds, particularly because of their quite peculiar bonding patterns, beginning with the simplest hydrides. Diborane was synthesized for the first time in the XIX century but investigated for the first time in the thirties of the XX century by Stock.^6^ We had to wait until the middle of the XX century to have a clear idea of its structure^7^ stabilized through the formation of three-center two-electron (3c–2e) bonds^8,9^ that lately would be found in many other systems, such as carboranes,^10^ trihydrogen cation (H_3_^+^),^11^ and, very recently, in ammonia triborane.^12^ A nice summary of this beautiful chapter in the history of chemistry can be found in ref (13). Rather interestingly, although diborane is a stable molecule, triborane(9) was found to be an unstable intermediate in the pyrolysis reaction of diborane.^14,15^ Later, B_3_H_9_ was described as a transient cyclic *C*_3*v*_ species,^16^ though more recently another non-cyclic *C*_2_ structure of triborane(9) was reported in which the central B atom appeared pentacoordinated.^17^ When moving to aluminum hydrides, dialane(6) is already a challenging derivative initially characterized through ab initio calculations.^18,19^ These theoretical predictions helped to identify this molecule in 2003 by seven of its infrared absorptions in solid hydrogen.^20^ Unexpectedly, however, Al_4_H_6_ was found to be a very stable system^21^ that motivated the study of Al_*n*_H_*n*+2_ (4 ≤ *n* ≤ 8) aluminum hydrides.^22^ A few years before, Kawamura et al.^23^ had identified different isomers for (AlH_3_)_*n*_ (*n* = 3–7) clusters, predicting for trialane(9) two isomers, a cyclic and an open one, the latter also showing a central pentacoordinated Al, exactly alike the ones described for triborane(9) back in 1995.^17^

Diborane halides, namely B_2_F_6_ and B_2_Cl_6_, are apparently not formed in the gas phase,^24^ whereas this is not the case for Al_2_F_6_ and Al_2_Cl_6_. The infrared spectrum of Al_2_F_6_ registered by matrix isolation was reported by Snelson,^25^ whereas Al_2_Cl_6_ can be obtained by vaporization of stable AlCl_3_ ionic crystals,^26^ and its structure was determined by gas-phase electron diffraction.^27^ The structure and bonding of both dimers were described using a minimal basis set^28^ and more recently through the use of B3LYP/Def2-TZVPP calculations showing that the dissociation energy for Al_2_F_6_ is greater than that of Al_2_Cl_6_.^24^

Very little attention has been paid to mixed clusters of B and Al. We are aware of the theoretical study of the structure and stability of aloborane(6),^29,30^ but we could not find similar studies on mixed dimers involving BF_3_ and AlF_3_ or BCl_3_ and AlCl_3_.

The motivation of this study is to provide a first description of the characteristics and stabilities of these mixed clusters in the gas phase, and of the effects that the interaction with a third monomer will bring about when the corresponding homo- and heterotrimers are formed. To achieve these goals, we present a systematic study, using different theoretical formalisms, of the dimers and trimers involving BX_3_ and AlX_3_ (X = H, F, Cl). As mentioned above, for X = H, the dimers and some of the trimers have already been discussed in the literature and will be used as a reference to verify the reliability of our calculations and to highlight the structural differences with the corresponding halides.

## Methods

To accurately calculate the thermodynamic stability of the dimers included in this study, we first considered the use the Gaussian-4 (G4) theory.^31^ In this procedure, the final energies are calculated by combining contributions obtained through the use of Møller–Plesset (MPn) perturbation theory up to the fourth order and CCSD(T) coupled cluster theory to properly account for the electron correlation effects. To these MPn and CCSD(T) energy contributions, an estimation of the Hartree–Fock energy limit (HFlimit) together with two high-level empirical corrections are added, in order to ensure that the final energies are accurate up to a CCSD(T,full)/G3LargeXP + HF limit level. The result is that G4 provides energetic outcomes for different thermodynamic properties of a large set of chemical compounds with an average absolute deviation^31^ of 3.47 kJ·mol^–1^. It must be taken into account, however, that the aforementioned ab initio calculations in the standard G4 procedure are carried out on previously B3LYP/6-31G(2df,p) optimized geometries. Since, as we shall discuss later, dispersion is an important component of the interaction energy in many of the clusters investigated in this paper and it is not correctly described at the B3LYP level, we will also use MP2/aug-cc-pVTZ optimized geometries in our G4 calculations, which will be referred hereafter as G4b results.

Unfortunately, the G4 formalism is too expensive to evaluate the trimers, in particular those involving Al and Cl. Hence, for all the systems, in order to have a uniform accuracy, we decided to carry out M06-2X/aug-cc-pVTZ calculations because it is known that this method yields values that correlate very well with the MP2 ones, in particular when an extended basis set is used.^32−35^ The M06-2X functional also provides descriptions of dispersion-dominated systems of much better quality than standard functionals.^36^ To check the relevance of dispersion for some of the clusters under investigation, at an affordable computational price, we have included in the computational study the B3LYP method adding the D3BJ empirical dispersion term proposed by Grimme including the Becke–Johnson damping correction.^37^

The bonding characteristics of the clusters under scrutiny will be analyzed through the use of four complementary procedures, namely, the atoms in molecules (AIM) theory,^38^ the electron localization function (ELF) formalism,^39^ the NCIPLOT approach,^40^ and the adaptive natural density partitioning (AdNDP) analysis.^41^ The AIM method is based on a topological analysis of the molecular electron density, ρ(*r*), by locating its critical points, in particular the first-order saddle points, named bond critical points (BCPs), commonly associated with the existence of a bonding interaction. This procedure also provides useful structural information through the so-called molecular graph that allows to confirm the formation of ring structures. All these calculations have been carried out by using the AIMAll (Version 19.10.12) code.^42^ The ELF approach^39^ analyzes the probability of finding, for a given chemical system, an electron in the same position as a reference electron with the same spin. This leads to the definition and classification of the areas in which the electrons of the system are distributed in monosynaptic and disynaptic (or polysynaptic) basins. The monosynaptic basins are associated with a single nucleus and correspond to core electrons and/or electron lone pairs. Conversely, the disynaptic (or polysynaptic) basins correspond to two-center or more than two-centers bonding regions. The NCIPLOT^40^ allows finding regions of low reduced density gradient (*s*) and low density values associated to noncovalent interactions. In the 2D diagrams, the RDG is represented toward the sign of the second eigenvalue of the Hessian (λ_2_) multiplied by the electron density, revealing peaks that account for the interactions, whereas in the 3D representations the interactions can be visualized in the real space as isosurfaces. The distinction between attractive or repulsive interactions, as well as their intensity, is facilitated through a red-green-blue range of colors. Finally, the use of the AdNDP analysis^41^ will allow us to obtain a complementary description of the chemical bonding of the systems under investigation, in particular the possible multicenter character of some of the bonds involved in them.

To analyze some of the characteristics of the clusters under scrutiny, in particular the trimers, it is convenient to evaluate the one-, two-, and three-body contributions to the binding energy. This has been done in the framework of the many-body interaction-energy (MBIE) formalism.^43,44^ For a ternary complex, the binding energy Δ*E* (eq 1) can be decomposed into one- (eq 2), two- (eq 3), and three-body interactions (eq 4), as follows:
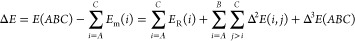
1
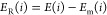
2
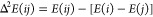
3
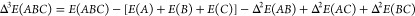
4The value of *E*_R_(*i*), the monomer distortion energy, is the difference between *E*_m_(*i*), the energy of the *i*-monomer in its equilibrium geometry, and *E*(*i*), the energy of the *i*-monomer within the geometry of the ABC complex. Δ^2^*E*(*ij*) and Δ^3^*E*(*ABC*) are the two- and three-body interaction energies computed at the corresponding geometries in the complex. We have also carried out an energy decomposition analysis based on the generalized Kohn–Sham and the localized molecular orbital energy decomposition analysis (LMO–EDA)^45^ in which the interaction energy is given by eq 5
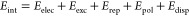
5where the first term describes the classical Coulombic interaction between the occupied orbitals of the monomers, the second to the third terms are the exchange and repulsive components associated with the Pauli exclusion principle, and the fourth and the fifth account for polarization and dispersion effects. Note that the LMO–EDA interaction energy differs from the MBIE one, because in the former, the distortion energy is not included. These LMO–EDA calculations were carried out by using the GAMESS code (version 2012-R1).^46^

As a first step of our analysis, we have carried out a screening for the different dimers and trimers using the CREST (Conformer–Rotamer ensemble sampling tool) method.^47,48^ We have found that the number of possible conformers is relatively high only in those clusters stabilized by van der Waals interactions (see Table S1 of the Supporting Information), but our discussion will focus exclusively on the most stable clusters.

## Results and Discussion

### BBX_6_, AlAlX_6_, and BAlX_6_ (X = H, F, Cl) Dimers

**Figure 1:**
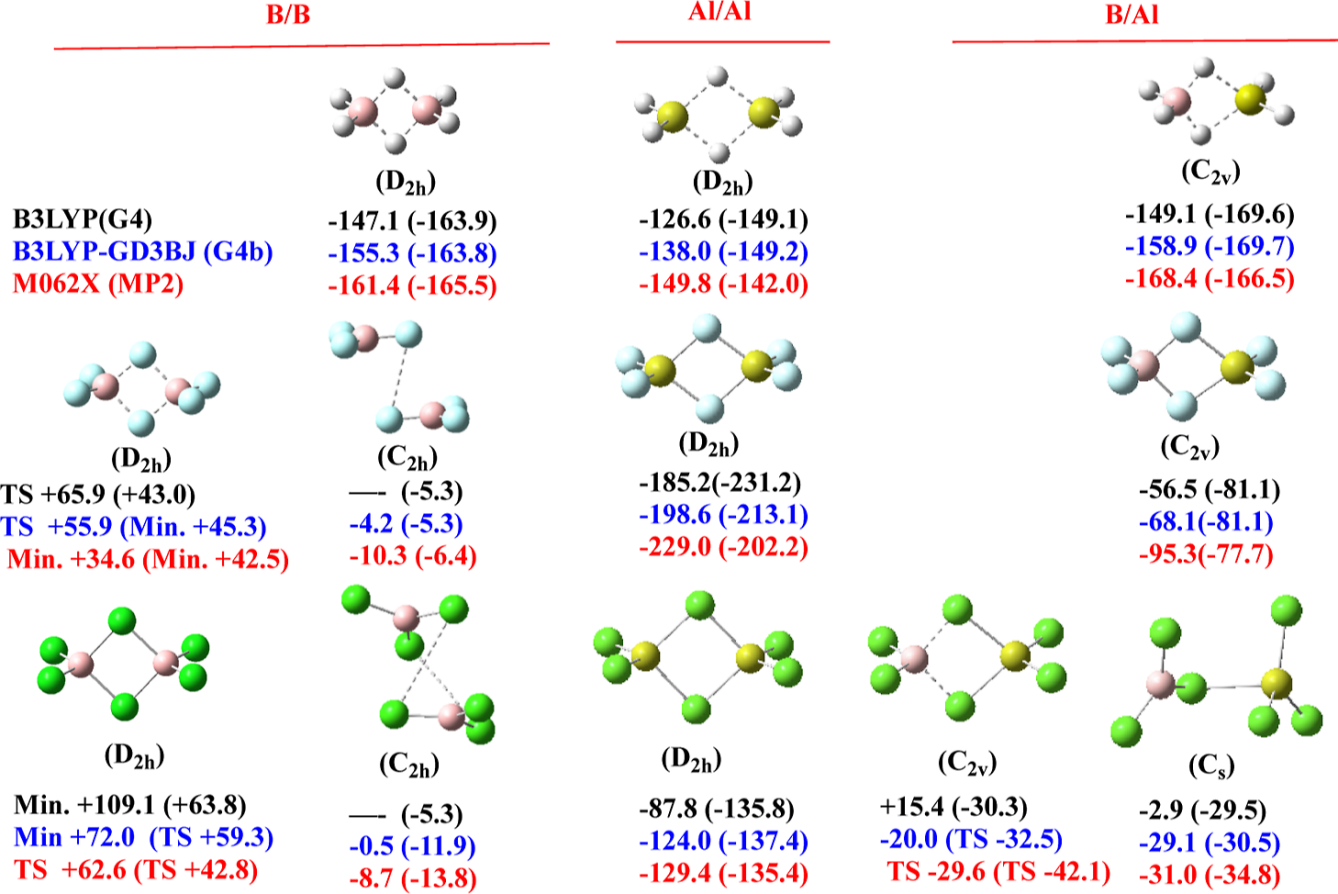
Structures (optimized at the MP2 level) of the BBX_6_, AlAlX_6_, and BAlX_6_ (X = H, F, Cl) dimers. Their dimerization enthalpies (in kJ·mol^–1^) with respect to the isolated monomers obtained with the indicated six theoretical schemes are also given.

In Figure 1, we present the structures of the different dimers investigated, indicating also the dimerization enthalpies (in kJ·mol^–1^) with respect to the isolated monomers obtained with the six different theoretical schemes summarized above. To start with, we should emphasize the excellent correlation found between all the M06-2X dimerization and trimerization enthalpies of the trimer hydrides (B_3_H_9_, Al_3_H_9_, B_2_AlH_9_, and BAl_2_H_9_) with respect to the G4b ones (H(M06-2X) = 0.9945 H(G4b) −2.254, *R*^2^ = 0.997, see Figure S1 of the Supporting Information), which ratifies the suitability of the M06-2X/aug-cc-pVTZ approach to describe this kind of clusters.

As mentioned in the Introduction, the triel hydride dimers have been reported before in the literature,^18,19,29,30^ so we are not going to discuss them in detail. All dimerizations are largely exothermic and we will only highlight that for diborane, our G4b dimerization energy at 0 K is only 2.9 kJ·mol^–1^ smaller than that obtained from the most recent total atomization energies at 0 K reported by Karton and Martin using the W4 theory,^49^ and that our results show that the dimerization enthalpies follow the trend AlBH_6_ > B_2_H_6_ > Al_2_H_6_, in agreement with previous studies.^30^

Something analogous can be said with respect to the Al_2_X_6_ (X = F, Cl) dimers, for which our results are similar to those of previous studies in the literature and show the large stability of the dialane-like pattern.^24^ A different matter is the question of the boron-containing analogues B_2_F_6_ and B_2_Cl_6_, which are believed to not exist.^24^ Indeed, the diborane-like *D*_2*h*_ structures for these dimers (first column in Figure 1) are found to be much higher in energy than the two isolated monomers, no matter the theoretical procedure used (see Figure 1). However, the *C*_2*h*_ isomers (second column in Figure 1) are not stable at the B3LYP level, as theoretically described in previous works,^50^ but they are found to be stable species, though weakly bound, when the methods used account for dispersion interactions. This is also consistent with the LMO–EDA analysis (see Table S2 of the Supporting Information) that shows that whereas for all the dimers included in Figure 1, the dispersion contribution to the total interaction energy is residually small (never greater than 8%), for B_2_F_6_ and B_2_Cl_6_, it is significantly large and the most important attractive contribution (42 and 58%, respectively). The dispersion contributions are smaller in the mixed BAlF_6_ dimer, but still significant in BAlCl_6_. As a matter of fact, as shown in Figure 1, the *C*_2*v*_ structure is predicted to be a minimum when the dispersion contributions are not accounted for, whereas if the dispersion is included it becomes a transition state, the global minimum being the *C*_*s*_ dimer with a dispersion contribution of 24%.

**Figure 2:**
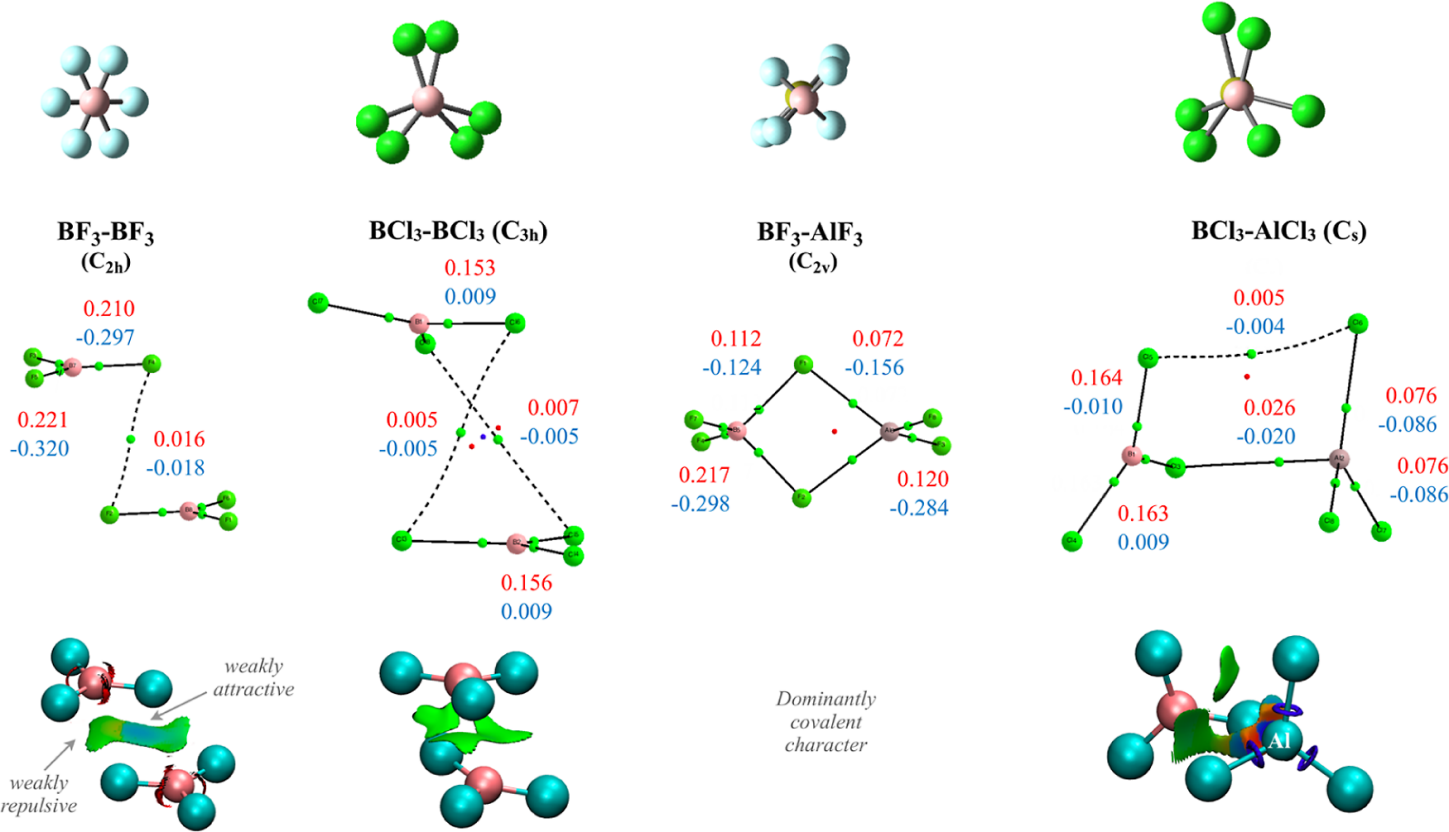
First row: eclipsed B–B, B–Al views to appreciate the halogen relative positions according to the different symmetries. Second row: molecular graphs of B_2_F_6_, B_2_Cl_6_, BAlF_6_, and BAlCl_6_ global minima, showing the electron density (red) and its Laplacian (blue) (a.u.) at the corresponding BCPs (green dots); ring CPs (in red) and cage CPs (blue) are also shown. Third row: 3D representations of noncovalent interactions obtained with NCIPLOT (*s* = 0.5, 0.3, and 0.3, respectively), color code: red (strongly repulsive), green (weakly attractive and weakly repulsive), and blue (strongly attractive).

This is also confirmed by the characteristics of the analysis of the topology of the electron density through different methods. The AIM molecular graphs of B_2_F_6_, B_2_Cl_6_, BAlF_6_, and BAlCl_6_ (second row in Figure 2) evidence that the B_2_F_6_ and B_2_Cl_6_ are not stabilized, as expected, by B···F or by B···Cl interactions, but by dispersive interactions between halogen atoms of different monomers, characterized by a very small electron density and its Laplacian at the corresponding BCP. The characteristics found for these F···F closed shell interactions are totally similar to those reported in previous works in different chemical environments.^51−53^ As usual in typical dispersion-governed systems, as for instance the methane dimer,^54^ both exhibit a continuous *s* isosurface associated to a region of weak interactions placed between monomers (third row in Figure 2), where not only the weakly attractive interactions but also the very weakly repulsive forces are revealed in a subtle balance. The ELF pattern of both dimers is very similar (see Figure S2); the only difference is a larger F lone pair population with respect to Cl, in agreement with a softer distribution of the electron density of the latter one and in line with its more complex AIM molecular graph.

We have considered it of interest to check weather these characteristics are likely to be preserved in solution. Hence, we have calculated, at the M062X/aug-cc-pVTZ level, the structure and stability of the fluorine and chlorine containing dimers in water solution using a polarizable continuum model.^55^ The results obtained (see Figure S3 of the Supporting Information) show that neither the structures nor the stabilities change dramatically with respect to the values obtained in the gas phase and shown in Figure 1. For the sake of completeness, we have also included in Figure S3 the cyclic BF_3_ trimers, where a similar situation was found (vide infra) with analogous results. It must be emphasized that these preliminary results in solution must be taken with caution, because a thoughtful investigation of solvent effects would require a more sophisticated model, including both specific and bulk solvation effects, which is clearly beyond the scope of this paper.

The *C*_2*v*_ form of the BAlF_6_ dimer is instead stabilized by B···F···Al linkages, in agreement with a rather small contribution of dispersion, whereas the *C*_*s*_ form of the BAlCl_6_ dimer is an intermediate situation with a B···Cl···Al linkage and a Cl···Cl dispersive interaction, in agreement with an intermediate LMO–EDA dispersion contribution. Indeed, the corresponding reduced density gradient isosurfaces show additional Cl···Cl interactions. It should be noted that the molecular graph of BAlF_6_ exhibits straight bond paths along the B···X···Al three-center bonds, similar to that found for bifurcated triel bonds,^56^ unlike the curved paths characteristic of the unique behavior of hydrogen.

**Figure 3:**
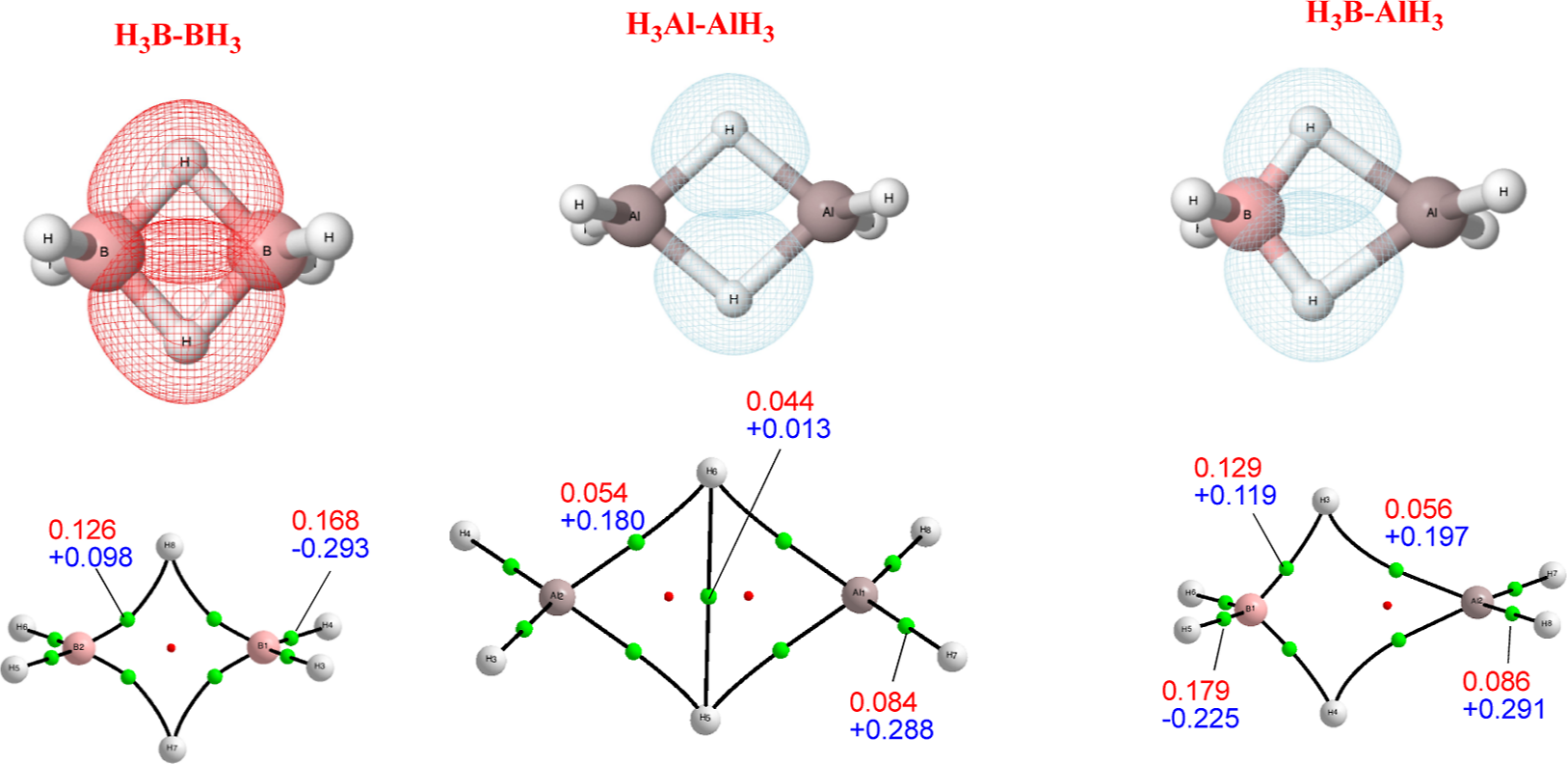
First row, AdNDP 3c–2e MOs for diborane, dialane, and H_3_B–AlH_3_ mixed-dimer. Second row, molecular graphs for the same systems, showing the electron density (red) and its Laplacian (blue) (a.u.) at the corresponding BCPs. Red and blue colors are used for MOs involving, exclusively, B and for MOs involving Al, respectively.

At this point, it would also be interesting to analyze whether in these dimers, the typical 3c–2e bonds that stabilize diborane^57^ are also present, like in the other dimers. The AdNDP analysis, which is a suitable tool to detect the formation of multicenter bonds,^58,59^ shows (see Table S3 of the Supporting Information and Figure 3) the well-known formation of two B···H···B 3c–2e bonds. The situation is similar for dialane but not identical since the corresponding orbital is more localized on the H atom of the bridge than in diborane (see Figure 3). This is a consequence of the much lower electronegativity of Al. Indeed, whereas the ∇^2^ρ at the non-bridging B–H BCPs is negative, as it should be expected for covalent interactions, for the Al–H bonds ∇^2^ρ is positive indicating a non-negligible ionic character in these bonds. Consequently, whereas the natural charge at the H bridge in diborane is slightly positive +0.11 e), in dialane, it is significantly negative (−0.37 e). This is also coherent with the appearance of a BCP between the two bridge hydrogens. Some peculiarities are also observed for the mixed dimer, H_3_B–AlH_3_. The AdNDP analysis also found a 3c–2e interaction, but the population is strongly localized in the B–H region where, 1.7 e^–^ of the bonding pair are localized in it, the remaining 0.3 e^–^ being located at the Al–H region. The molecular graph still is coherent with a 3c–2e bond but, as indicated by the AdNPD, strongly polarized toward the B.

The situation is ambiguous when dealing with the F and Cl derivatives. As shown in the lower part of Table S3 of the Supporting Information, for the BF_3_–AlF_3_ dimer, a model including 3c–2e bonds has a number of residual electrons (0.83) larger than the conventional model with only 2c–2e bonds (0.61). For the BCl_3_–AlCl_3_ dimer, this difference is much larger (1.39) for a 3c–2e description vs (0.89) for the conventional model, hence these clusters seem to be more appropriately described by a 2c–2e model, with the orbitals significantly polarized by the Al positive charge.

### Homotrimers

**Figure 4:**
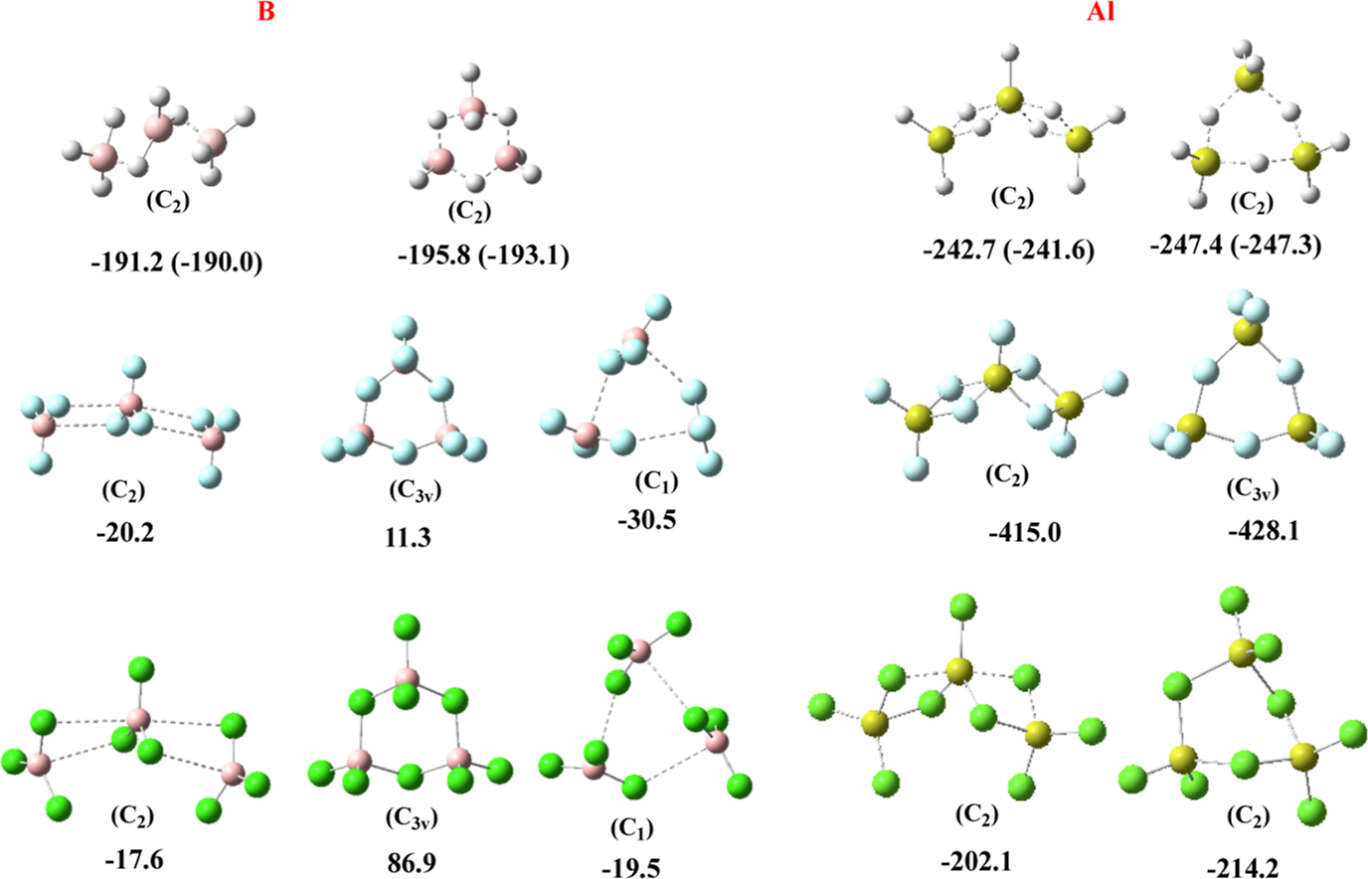
Structures of the B_3_X_9_ and Al_3_X_9_ (X = H, F, Cl) trimers, with their M06-2X trimerization enthalpies (in kJ·mol^–1^) with respect to the isolated monomers (values in parenthesis were obtained at the G4b level of theory).

The structures and trimerization enthalpies of B_3_X_9_ and Al_3_X_9_ (X = H, F, Cl) are shown in Figure 4 at the M06-2X/aug-cc-pVTZ level of theory.

**Figure 5:**
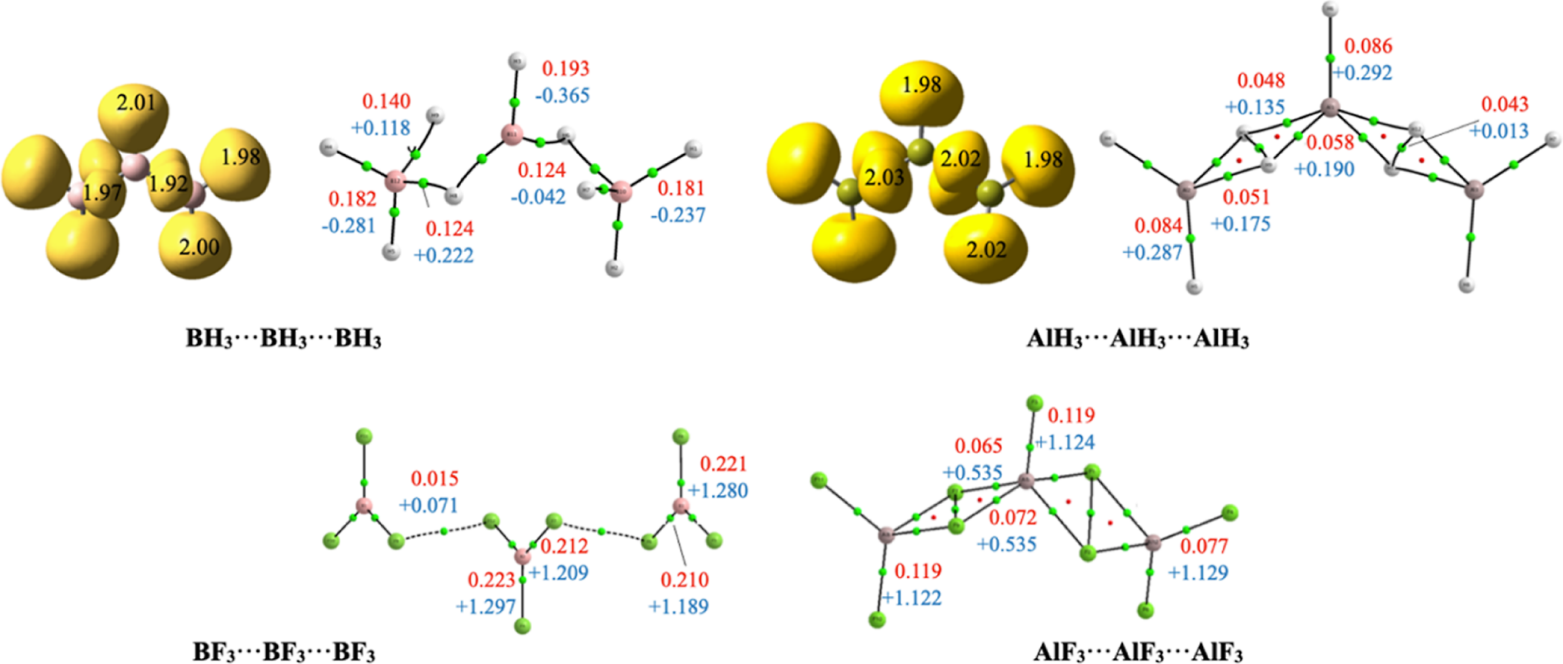
First row: ELF basins and molecular graphs of B_3_H_9_ and Al_3_H_9_. Second row: molecular graphs of B_3_F_9_ and Al_3_F_9_. The molecular graphs show the electron density (red) and its Laplacian (blue) (a.u.) at the corresponding BCPs (green dots); ring CPs in red are also shown. ELF (0.8) disynaptic and trisynaptic basins involving H atoms are colored in yellow. Populations are shown in atomic units (e).

As for the case of the dimers, the hydride trimers had already been described in the literature,^17,23^ showing again very large stabilization enthalpies. We want to call the attention of the reader to the fact that the linear triborane(9) was described as a *C*_2_ isomer with a pentacoordinated central boron atom. However, the topological analysis in Figure 5 shows no BCPs connecting the two most distant (1.44 Å) B and H atoms, and only 3 BCPs with two curve paths (B–H distance 1.35 Å) surround the central B atom; conversely, five BCPs are found around Al in the *C*_2_ linear structure of the Al-containing analogue, in line with a covalent pentacoordinated pattern. It should be noticed that whereas the ∇^2^ρ for the B–H bonds is negative, as it should be expected for covalent interactions, for the Al–H bonds ∇^2^ρ, it is positive indicating a non-negligible ionic character in these bond, which becomes even higher in Al–F linkages (see Figure 5).

Nonetheless, the ELF partition of the molecular space in both B_3_H_9_ and Al_3_H_9_ is pretty alike, finding one disynaptic (T, H) basin and four trisynaptic (T, H, T) basins around the central triel (T = B, Al) atoms. For both compounds, the four trisynaptic basins are remarkably polarized and smaller than the rest, containing approximately eight electrons in both cases. It looks like the electronic cloud around these two more distant hydrogens in the boron trimer is too compact to lead to two BCPs; in fact, the basin volumes are only 59 u^3^, compared to 107 u^3^ for Al. As we will see later, the clearly pentacoordinated pattern of Al seems to be a systematic behavior in all the trimers we have investigated; in this sense, it is interesting to note that whereas diborane(6) is more stable than dialane(6) (−161 vs −150 kJ·mol^–1^), trialane(9) is significantly more stable than triborane(9) (−243 vs −191 kJ·mol^–1^). Due to this enhanced stability, whereas the dissociation of B_3_H_9_ into B_2_H_6_ + BH_3_ requires an energy of 29.8 kJ·mol^–1^, a similar process for Al_3_H_9_ requires more, three times this value (93.0 kJ·mol^–1^).

This stability difference is huge when comparing the B_3_F_9_ and Al_3_F_9_ linear *C*_2_ trimers, similar to what is observed for the dimers on going from the hydrides to the halides. As shown in the molecular graphs in Figure 5, binding in B_3_F_9_ is again based on F···F dispersive interactions and the central B atom is tricoordinated. Conversely, the Al_3_F_9_ trimer shows a pentacoordinated central Al atom and a stability much larger than the B containing analogue. The situation is totally similar when B_3_Cl_9_ is compared with the Al_3_Cl_9_ analogues (see Figure S4 of the Supporting Information). As a consequence, whereas the dissociation of Al_3_F_9_ into Al_2_F_6_ + AlF_3_ (Al_3_Cl_9_ into Al_2_Cl_6_ + AlCl_3_) requires an energy of 186.0 (72.5) kJ·mol^–1^, the same process for B_3_F_9_ (B_3_Cl_9_) requires only 10.0 (8.9) kJ·mol^–1^. Consistently, the LMO–EDA analysis shows that the dispersion contribution in Al_3_F_9_ (Al_3_Cl_9_) accounts only for the 10% (13%) of the total energy, in contrast with a 43% (58%) in B_3_F_9_ (B_3_Cl_9_) (see Table S4 of the Supporting Information). Because of the dispersive stabilization of the B_3_F_9_ (B_3_Cl_9_) trimer, the standard B3LYP or G4 formalisms fail to find it, hence the only way to apply the G4 formalism to describe this kind of clusters is by using MP2 rather than B3LYP optimized geometries. With the exception of the homotrimers involving BF_3_ and BCl_3_ that, as discussed above, are stabilized by dispersion interactions, the AdNDP analysis shows that 3c–2e patterns similar to those discussed above for the dimers are present in the H_*m*_B–BH_*n*_ subunits forming the (BH_3_)_3_ trimer (vide infra). The same can be said with respect to the X_*m*_Al–AlX_*n*_ (X = H, F, Cl) subunits forming the (AlX_3_)_3_ trimers.

There is still another interesting result in Figure 4 that requires to be commented. Whereas the *C*_2_, *C*_3*v*_, and *C*_2_ cyclic structures of B_3_H_9,_ Al_3_F_9_, and Al_3_Cl_9_, respectively, are found to be the global minima of these ternary complexes, the similar *C*_3*v*_ structures for B_3_F_9_ and B_3_Cl_9_ trimers are not since they are 11 and 87 kJ·mol^–1^ less stable than the three isolated monomers. Also, surprisingly, these two species have significantly strong bonds as compared with those stabilizing the *C*_1_ cyclic global minima. Indeed, whereas the *C*_1_ global minima are stabilized through weak F···F and Cl···Cl dispersive interactions (contribution of the dispersion energy 57% of the total energy), the *C*_3v_ isomers exhibit quite strong B–F and B–Cl bonds, as reflected by dispersion contributions of only a 10% large electron density and highly populated ELF basins typical of strong covalent bonds (see Figures S5 and S6 of the Supporting Information). Hence, the question is why are they unstable with respect to the isolated monomers? To have an answer, it is necessary to employ the MBIE analysis to evaluate the one-, two-, and three-body contributions to the binding energy, which is summarized in Table 1 for the aforementioned ternary complexes.

**Table 1:** MBIE Analysis of the Ternary Complexes Formed by BH_3_, BF_3_, and BCl_3_^a^

ternary complex	*E*_R_(*A*)	*E*_R_(*B*)	*E*_R_(*C*)	Δ^2^*E*(*AB*)	Δ^2^*E*(*AC*)	Δ^2^*E*(*BC*)	Δ^3^*E*(*ABC*)	*E*_total_
B_3_H_9_ (*C*_2_, linear)	61.3	65.1	61.3	–199.3	–16.8	–199.3	7.6	–220.1
B_3_H_9_ (*C*_2_, cyclic)	117.4	116.4	116.7	–206.4	–198.9	–206.1	34.0	–226.9
B_3_F_9_ (*C*_2_, linear)	0.6	0.5	0.6	–15.5	–0.3	–15.5	0.0	–29.6
B_3_F_9_ (*C*_3*v*_, cyclic)	164.6	164.6	164.6	–107.3	–107.3	–107.3	–165.6	6.3
B_3_F_9_ (*C*_1_, cyclic)	0.6	0.5	0.6	–15.5	–0.3	–15.5	0.0	–29.6
B_3_Cl_9_ (*C*_2_, linear)	0.1	0.1	0.1	–13.6	–0.5	–13.6	0.3	–27.3
B_3_Cl_9_ (*C*_3*v*_, cyclic)	137.3	137.3	137.3	–65.0	–65.0	–65.1	–135.9	81.1
B_3_Cl_9_ (*C*_1_, cyclic)	0.1	0.1	0.0	–8.0	–7.9	–10.8	–2.8	–29.3

a All values in kJ·mol^–1^.

In view of this table and recalling eq 1, it is apparent that the dominant positive terms for all cyclic trimers correspond to the monomer distortion energies, *E*_R_(*i*), and only in few cases, the three-body term Δ^3^*E*(*ABC*) is marginally positive. For all the linear clusters, with the only exception of the *C*_2_ linear triborane, these monomer distortion energies are negligibly small. However, whereas the distortion energies in cyclic triborane are compensated by the large stability of the B–H bonds formed, the large stability of the new B–F and B–Cl bonds in cyclic B_3_F_9_ and B_3_Cl_9_ (see Figures S4 and S5 of the Supporting Information) is not enough to compensate the high values of the *E*_R_(*i*) components, resulting in a positive total energy. The high monomer distortion should come essentially from the rehybridization suffered by B or Al to go from a totally planar conformation in the isolated monomer to a tetrahedral conformation in the corresponding ternary complex. It is apparent that BF_3_ presents the largest distortion energy, followed by BCl_3_ and BH_3_. We have calculated, at the M06-2X/aug-cc-pVTZ level used in our work, the energy cost to go from the equilibrium conformation of these three monomers (X–B–X angle = 120°) to a conformation in which the X–B–X angle −is the tetrahedral one (≈109.5°). The values found (in kJ·mol^–1^: 94 for BH_3_, 169 for BF_3_, and 140 for BCl_3_) not only follow the same trend as the *E*_R_(*i*) values in Table 1 but are so close to them that they allow us to conclude that the rehybridization of B (and Al) is the fundamental contributor to the monomer distortion and the energetic cost behind the instability of the *C*_3*v*_ cyclic structures of B_3_F_9_ and B_3_Cl_9_ trimers.

### Heterotrimers

**Figure 6:**
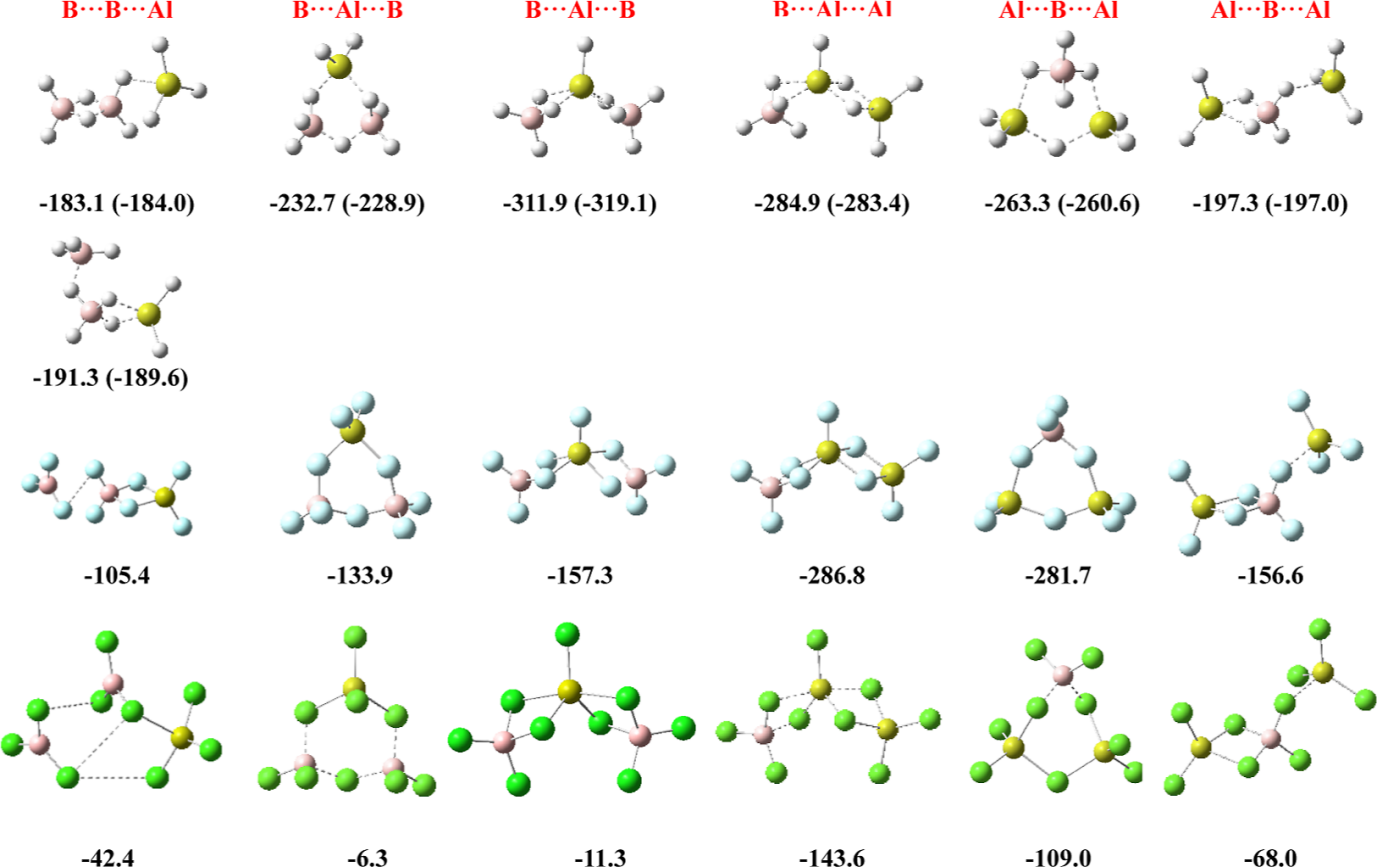
Structures of the B_2_AlX_9_ and BAl_2_X_9_ (X = H, F, Cl) ternary complexes, with their M06-2X trimerization enthalpies (in kJ·mol^–1^) with respect to the isolated monomers (values in parenthesis were obtained at the G4b level of theory).

The M06-2X/aug-cc-pVTZ structures and the stabilization enthalpies (kJ·mol^–1^) of the B_2_AlX_9_ and BAl_2_X_9_ (X = H, F, Cl) trimers with respect to the isolated monomers are shown in Figure 6.

**Figure 7:**
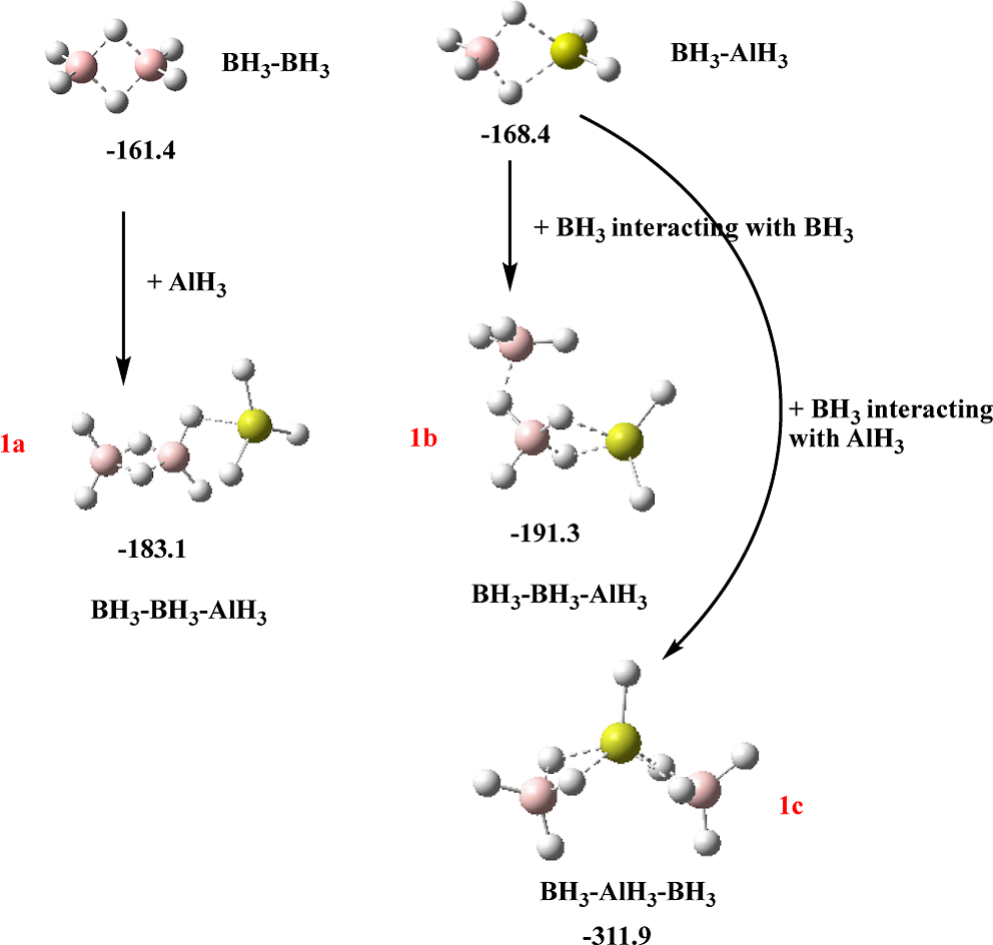
Schematic representation of the possible mechanisms associated with the formation of isomers **1a**, **1b**, and **1c** of B_2_AlH_9_ trimers. The stabilization enthalpies are in kJ·mol^–1^.

The results obtained for the mixed hydrides indicate, once again, large stabilization energies in all cases. The systems with the highest values are controlled by the ability of the central Al to be pentacoordinated, a coordination pattern never observed for B in central positions. Notably, the most stable isomer exhibits a linear B–Al–B arrangement more stable than the corresponding cyclic one (−312 vs −232 kJ·mol^–1^). The two B–B–Al arrangements are clearly less favorable (−183, −191 kJ·mol^–1^). These results can be understood with the help of the scheme presented in Figure 7. Indeed, the isomers with a B–B–Al sequence can be obtained by attaching (i) AlH_3_ to diborane or (ii) BH_3_ to the BH_3_ unit of the mixed dimer BH_3_–AlH_3_.

The first mechanism leads to the **1a** isomer, whereas the second yields a more stable isomer **1b**. The abovementioned most stable linear isomer B–Al–B (**1c**) can be obtained attaching BH_3_ to BH_3_–AlH_3_ through Al.

**Figure 8:**
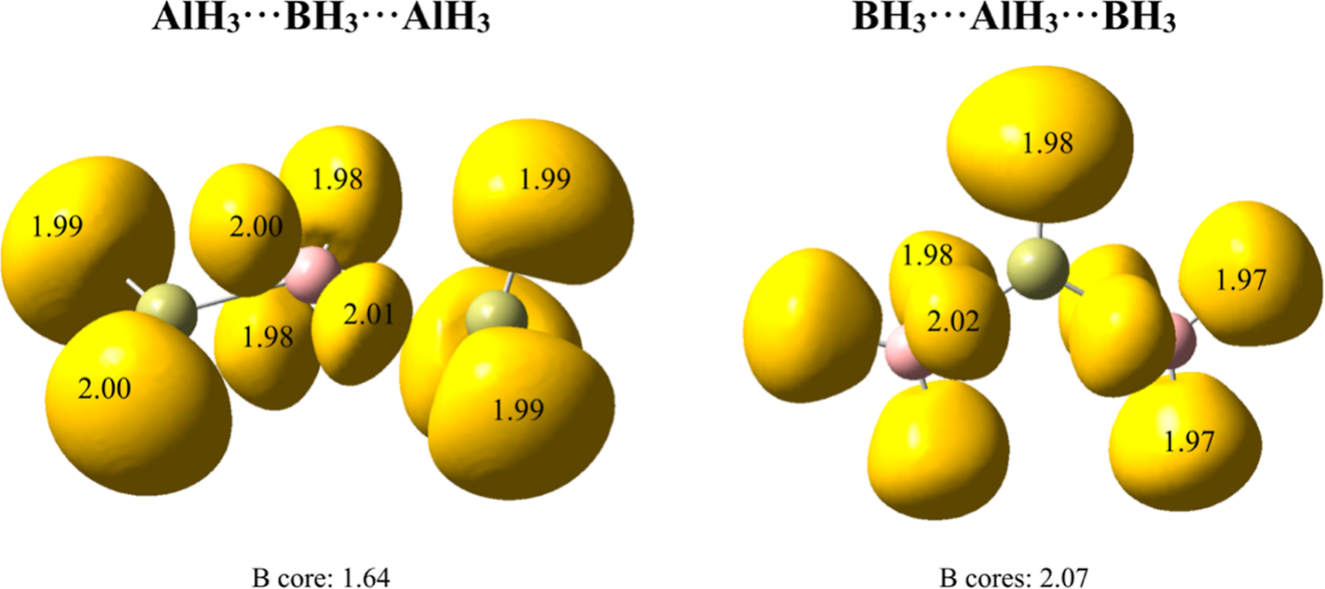
ELF basins of structures BAl_2_H_9_ (isomer −197.3 kJ·mol^–1^ in Figure 6) and B_2_AlH_9_ (isomer −311.9 kJ·mol^–1^ in Figure 6, **1c** in Figure 7). ELF (0.8) disynaptic and trisynaptic basins involving H atoms are colored in yellow. Populations are shown in atomic units (e).

The characteristics of trimers (**1a–c**) are clearly reflected in the corresponding molecular graphs (see Figure S7 of the Supporting Information). Coherently with the AIM description, the AdNDP analysis shows that in **1a** and **1b,** there is only one 3c–2e bond between the central BH_3_ group and the AlH_3_ group in the former and between the central BH_3_ group and the external BH_3_ group in the latter, whereas in **1c**, the Al is pentacoordinated, forming four 3c–2e bonds with the two BH_3_ groups (see Figure S7 of the Supporting Information). Also, consistently, the ELF partition for this minimum presents five basins around Al (see Figure 8), one disynaptic (Al, H) and four trisynaptic (B, H, Al), as already observed for homotrimer Al_3_H_9_.

Notably, the mixed trimers with two AlH_3_ groups are quite different. As illustrated in Figure S8 of the Supporting Information, both mechanisms, association of BH_3_ to dialane or association of AlH_3_ to BH_3_–AlH_3_ through Al, lead to the most stable trimer with the central Al pentacoordinated. The association of AlH_3_ to BH_3_–AlH_3_ through B yields the less stable trimer (AlH_3_–BH_3_–AlH_3_) where B is only tetracoordinated, with only four basins around it, as illustrated in Figure 8. The AdNDP shows that these four basins correspond to one B–H 2c–2e bond and three B–Al 3c–2e bonds, so that one of the Al centers participates in only one 3c–2e bonds (see Figure S9). Rather interesting is the bonding in the BH_3_AlH_3_AlH_3_ trimer. The first conspicuous fact is that one of the H atoms of the central AlH_3_ group has been transferred to the BH_3_ one, so this subunit is actually a BH_4_ one. The AdNDP analysis shows that the central Al atom is hexacoordinated forming, besides the 2c–2e Al–H, three 3c–2e bons with the BH_4_ moiety and two more with the second AlH_3_, which apparently is not in agreement with the corresponding molecular graph (see Figure S9 of the Supporting Information), since it only shows a BCP between Al and B. However, the AdNDP what actually shows is a large concentration of electrons on the BH_4_ moiety that actually has a net charge of −0.69 e. Hence, this structure can be also viewed as a result of the interaction of a very positive Al atom (net charge +1.19) with an almost BH_4_ anion, description that will be coherent with AIM results and not in contradiction with the AdNDP description.

Regarding the halides, the characteristics of the mixed trimers are marked by the same weak dispersive interactions between BF_3_ groups and BCl_3_ groups already discussed for the dimers. This is evident for the BF_3_–BF_3_–AlF_3_ trimer that can be described as the result of a dispersive interaction of BF_3_ with the BF_3_ group of the BF_3_–AlF_3_ dimer (see Figure S10 of the Supporting Information). Consequently, its dissociation into BF_3_ + BF_3_–AlF_3_ requires only 38.7 kJ·mol^–1^ and the LMO–EDA contribution of dispersion is 12%. The situation is similar for the BCl_3_–BCl_3_–AlCl_3_, whose dissociation enthalpy into BCl_3_ + BCl_3_–AlCl_3_ is 11.5 kJ·mol^–1^ and the LMO–EDA dispersion contribution amounts to 31% of the total energy (see Table S5 of the Supporting Information). In line with this, their total stabilization energies are practically additive. In other words, since the interaction of the BF_3_ monomer with the BF_3_–AlF_3_ is weak, the stabilization enthalpy of the trimer formed (−105.4 6 kJ·mol^–1^) is practically equal to the sum of the stabilization enthalpies of the two dimers (BF_3_–BF_3_ and BF_3_–AlF_3_) that can be identified in the trimer (−105.6 kJ·mol^–1^). The same applies to the BCl_3_–BCl_3_–AlCl_3_, whose stabilization enthalpy (−42.4 kJ·mol^–1^) is very close to the sum of the stabilization enthalpies of the two dimers BCl_3_–BCl_3_ and BCl_3_–AlCl_3_ (−39.7 kJ·mol^–1^).

It should be noted, however, that the additivity rule is not always fulfilled. The BX_3_–AlX_3_–BX_3_ (X = F, Cl) trimers have a central pentacoordinated Al atom (see Figures S5 and S6) where the stabilization enthalpy of both trimers is smaller than twice the stabilization of the dimer BX_3_–AlX_3_ (X = F, Cl) in the trimer. Once again, this is easily explained by means of the MBIE analysis shown in Table 2 comparing the B–Al–B and B–B–Al sequences. When the BF_3_BF_3_AlF_3_ cluster is formed, the ER monomer distortion energy of the new BF_3_ interacting with BF_3_–AlF_3_ is negligibly small (0.5 kJ·mol^–1^), whereas the distortion energies of the other two monomers of the BF_3_–AlF_3_ of the dimer within the trimer (67.9 and 150.8 kJ·mol^–1^) are very close to those of the isolated dimer (66.5 and 155.9 kJ·mol^–1^), explaining the additivity observed since the formation of the trimer results indeed in a very small distortion of the two interacting subunits. The situation is different if the trimer formed is BF_3_–AlF_3_–BF_3_. In this case, the ER monomer distortion energy of the new BF_3_ interacting with BF_3_–AlF_3_ is as big as that of the BF_3_ forming the dimer (132.2 kJ·mol^–1^), whereas the attractive two-body interactions in the trimer (−220 kJ·mol^–1^) are smaller than in the isolated dimer (−321 kJ·mol^–1^). Both effects, increasing of the distortion energy of one of the monomers and decrease of the attractive two-body interactions, explain why in this case the trimer is less stable than predicted from a pure additive scheme. In both examples, the results are similar when F is replaced by Cl (see Table 2). To finish, the BX_3_–BX_3_–AlX_3_ (X = F, Cl) complexes are not found with the standard B3LYP or G4 methods, as observed above because of the dispersion treatment.

**Table 2:** MBIE Analysis for the Linear Mixed BF_3_BF_3_AlF_3_, BF_3_AlF_3_BF_3_, BCl_3_BCl_3_AlCl_3_, and BCl_3_AlCl_3_BCl_3_ Ternary Complexes, and the BF_3_AlF_3_, BCl_3_AlCl_3_ Dimers from Which They can be Obtained by Association with BF_3_ or BCl_3_ Monomers^a^

	*E*_R_(*A*)	*E*_R_(*B*)	*E*_R_(*C*)	Δ^2^*E*(*AB*)	Δ^2^*E*(*AC*)	Δ^2^*E*(*BC*)	Δ^3^*E*(*ABC*)	*E*_total_
Ternary Complexes								
BF_3_BF_3_AlF_3_	67.9	150.8	0.5	–317.1	–2.5	–9.6	–4.1	–114.1
BF_3_AlF_3_BF_3_	132.2	62.1	132.1	–220.3	–10.4	–220.1	–40.4	–164.7
BCl_3_BCl_3_AlCl_3_	4.1	13.0	0.2	–52.2	–1.7	–12.8	–2.5	–51.9
BCl_3_AlCl_3_BCl_3_	92.4	36.8	92.3	–103.8	–3.8	–102.9	–29.4	–17.8
Dimers								
BF_3_AlF_3_	66.5	155.9	_	–321.4	_	_	_	–99.0
BCl_3_AlCl_3_	5.1	12.4	_	–53.2	_	_	_	–35.7

a All values in kJ·mol^–1^.

As for the halide homotrimers, the central B atom of the AlF_3_–BF_3_–AlF_3_ and the AlCl_3_–BCl_3_–AlCl_3_ heterotrimers is not pentacoordinated, but the central Al atom of the BF_3_–AlF_3_–BF_3_ and BCl_3_–AlCl_3_–BCl_3_ is (see Figure S11 of the Supporting Information).

We discussed for the halide homotrimers why boron cyclic structures with strong bonds were not even stable; the stability of some of the boron-containing cyclic heterotrimers deserves a similar attention. For instance, whereas the cyclic isomer of the B3F9 (Figure 4) was 11 kJ·mol^–1^ less stable than the isolated monomers, the mixed B_2_AlF_9_ cyclic cluster is predicted to be 133.9 kJ·mol^–1^ more stable than the isolated monomers. Conversely, whereas the trimerization enthalpy of Al_3_F_9_ homotrimer was found to be −428.1 kJ·mol^–1^, that of the mixed BAl_2_F_9_ cluster is only −281.7 kJ·mol^–1^. This can be understood, as before, comparing the MBIE analysis in Table S6 of the Supporting Information with the values in Table 1. A comparison between cyclic B3F9 and cyclic B_2_AlF_9_ evidences that one of the monomer distortion energies, *E*_R_(*C*), becomes much smaller than the other two, as it corresponds to an AlF_3_ group. Concomitantly, two of the two-body stabilization energies become larger in B_2_AlF_9_ than in B_3_F_9_, because they correspond to the B–Al interactions rather than to the B–B ones. Both facts together lead to a B_2_AlF_9_ cluster stable with respect to the corresponding monomers. The reasons behind the lower stability of the cyclic BAl_2_F_9_ with respect to the Al_3_F_9_ homotrimer follow similar arguments: the monomer distortion energy, *E*_R_(*C*), becomes much smaller than the other two, as it corresponds to a BF_3_ group, whereas the two-body interaction energies associated with B–Al interactions become less negative, resulting in an overall smaller stabilization of the heterotrimer. Similar arguments can be applied when F is replaced by Cl, explaining that the B_2_AlC_l9_ heterotrimer becomes more stable than B_3_Cl_9_ (−6.3 vs 86.9 kJ·mol^–1^), and BAl_2_Cl_9_ becomes less stable than Al_3_Cl_9_ (−109.0 vs −214.2 kJ·mol^–1^).

## Conclusions

Our results for triel hydride dimers and Al_2_X_6_ (X = F, Cl) clusters are in good agreement with previous studies in the literature, predicting for diborane a dimerization energy in close agreement with recent W4 total atomization energies,^49^ and the following trend AlBH_6_ > B_2_H_6_ > Al_2_H_6_ for the other hydride dimers, also in agreement with previous studies.^30^ However, and in contrast with the general accepted idea that B_2_F_6_ and B_2_Cl_6_ do not exist, we have found that these two dimers are weakly bound systems stabilized by dispersion interactions, so they cannot be found as stable species if dispersion is not accounted for in the theoretical schemes used. This is the case when a B3LYP functional is used, and, as a consequence, is also a failure of the standard G4 formalism because it is based on the B3LYP optimized geometries. According to our LMO–EDA analysis, in homo- and hetero-trimers, only the interactions of both BF_3_ and BCl_3_ groups with the rest of the cluster are dominated by dispersion. Another interesting result concerns B_3_F_9_ and B_3_Cl_9_ cyclic trimers of *C*_3*v*_ symmetry, because both clusters, in spite of exhibiting rather strong B–X (X = F, Cl) interactions, reflected in short interatomic distances and in the characteristics of their electron density distributions, analyzed by means of the AIM and NCIPLOT procedures, are not only less stable than the weakly bound *C*_1_ cyclic isomers but also unstable with respect to the isolated monomers. This rather surprising result is due to the high energetic cost of the rehybridization of the B atom to go from a planar B atom in the monomer to a tetrahedral B when the *C*_3*v*_ trimers are formed. On top of that, this monomer distortion energy is larger than the two- and three-body stabilization contributions when the cyclic cluster is formed, and, consequently, the trimerization energy becomes positive. Conversely, in the weakly bound *C*_1_ cyclic trimers, the two-body stabilization energies are much smaller than in the *C*_3*v*_ isomers, as it corresponds to trimers stabilized by dispersion interactions, but the distortion of the monomer within the trimer is negligible and the trimer becomes stable. Another important feature is the enhanced stability of both homo- and heterotrimers in which Al is the central atom with respect to those in which the central position is occupied by a B atom. Both the AIM and the NCIPLOT formalisms confirm that in the first case the Al is systematically pentacoordinated, whereas this is not the case when the central atom is B, which is only tri- or tetra-coordinated. Finally, from a more technical point of view we have found that, for a set including 21 different dimers and trimers, the correlation between G4b and M06-2X stabilization enthalpies is excellent, which suggests that the M06-2X method together with a flexible enough basis set may be a good alternative to study larger clusters.

## Supporting Information Available

Linear correlation between the M06-2X and the G4b stabilization enthalpies, the most stable conformers for (BH_3_)_2_(AlH_3_) and (BCl_3_)_2_(AlCl_3_) heterotrimers, the AdNPD orbital analysis for diborane, dialane, H_3_B–AlH_3_ mixed dimer and AlH_3_–BH_3_–AlH_3_ and of BH_3_–AlH_3_–AlH_3_, the LMO–EDA analysis for the BBX_6_, AlAlX_6_ and BAlX_6_ (X = H, F, Cl) dimers, the ELF basins of B_2_F_6_, B_2_Cl_6_ and BAlCl_6_ dimers and linear and cyclic B_3_F_9_ and B_3_Cl_9_ trimers, the structure and relative stability of some clusters in solution, the molecular graphs of linear B_3_Cl_9_ and Al_3_Cl_9_ clusters, cyclic isomers of B_3_X_9_ (X = H, F, Cl), B_2_AlH_9_, B_2_AlF_9_, and B_2_AlCl_9_, and AlX_3_BX_3_AlX_3_ (X = F, Cl), BX_3_AlX_3_BX_3_ (X = F, Cl), AlH_3_–BH_3_–AlH_3_ and of BH_3_–AlH_3_–AlH_3_ ternary complexes, and schematic representation of the possible mechanisms associated with the formation of BH_3_–AlH_3_–AlH_3_ isomers and the MBIE analysis for the cyclic mixed B_2_AlF_9_, BAl_2_F_9_, B_2_AlCl_9,_ and BAl_2_Cl_9_ ternary complexes (PDF)
