# Supplementary material for: Dispersion, Rehybridization, and Pentacoordination: Keys to Understand Clustering of Boron and Aluminum Hydrides and Halides
Source: J Phys Chem A. 2023 Jul 7;127(28):5860–71. doi: 10.1021/acs.jpca.3c02747 (PMC10364081; doi:10.1021/acs.jpca.3c02747)
Supplement: Supplementary file 1 — jp3c02747_si_001.pdf [file jp3c02747_si_001.pdf]

SUPPORTING INFORMATION

* * *

Dispersion, Rehybridization and Pentacoordination: Keys to Understand Clustering of Boron and Aluminum Hydrides and Halides

Otilia Mó[1], M. Merced Montero-Campillo[1]*, Manuel Yáñez[1]*, Ibon Alkorta[2]*, José Elguero[2].

[1] Departamento de Química, Módulo 13, Facultad de Ciencias, and Institute of Advanced Chemical Sciences (IAdChem), Universidad Autónoma de Madrid, Campus de Excelencia UAM-CSIC, Cantoblanco, 28049 Madrid, Spain.

[2] Instituto de Química Médica, IQM-CSIC, Juan de la Cierva, 3. 28006 Madrid, Spain.
mm.montero@uam.es, manuel.yanes@uam.es, ibon@iqm.csic.es

CONTENTS

Table S1. The most stable conformers for $(BH_3)_2(AlH_3)$ and $(BCl_3)_2(AlCl_3)$ heterotrimers.

Figure S1. Linear correlation between the M06-2X and the G4b stabilization enthalpies.

Table S2. LMO-EDA analysis for the BBX_6, $AlAlX_6$ and $BAlX_6$ (X = H, F, Cl) dimers.

Figure S2. ELF basins of B_2F_6, B_2Cl_6 and $BAlCl_6$ dimers

Figure S3. Structure and relative stability of some clusters containing F and Cl in water solution.

Table S3. AdNPD orbital list for diborane, dialane and H_3B-AlH_3 mixed dimer

Figure S4. Molecular graphs of linear B_3Cl_9 and Al_3Cl_9 clusters.

Table S4. LMO-EDA analysis for the $BBBX_6$ and $AlAlAlX_6$ (X = H, F, Cl) homotrimers.

Figure S5. Molecular graphs of global different cyclic isomers of B_3X_9 (X = H, F, Cl) ternary complexes.

Figure S6. ELF basins of linear and cyclic B_3F_9 and B_3Cl_9 trimers.

Figure S7. Molecular graphs and AdNDP 3c-2e MOs of B_2AlH_9 ternary complexes

Figure S8. Schematic representation of the possible mechanisms associated with the formation of BH_3-AlH_3-AlH_3 isomers.

Figure S9. Molecular graphs and AdNDP 3c-2e MOs for trimers AlH_3-BH_3-AlH_3 and of BH_3-AlH_3-AlH_3.

Figure S9. Molecular graphs of B_2AlF_9 and B_2AlCl_9 ternary complexes.

Table S5. LMO-EDA analysis for the $BBAlX_6$ and $BAlAlX_6$ (X = H, F, Cl) heterotrimers.

Figure S10. Molecular graphs of $AlX_3BX_3AlX_3$ (X = F, Cl) and $BX_3AlX_3BX_3$ (X = F, Cl) ternary complexes.

Table S6. MBIE analysis for the cyclic mixed B_2AlF_9, BAl_2F_9, B_2AlCl_9 and BAl_2Cl_9 ternary complexes.

Table S1. The most stable conformers for $(BH_3)_2(AlH_3)$ and $(BCl_3)_2(AlCl_3)$ heterotrimers selected through the use of the CREST formalism.

From the different conformers of the $(BH_3)_2(AlH_3)$ the 7 most stable were the following:

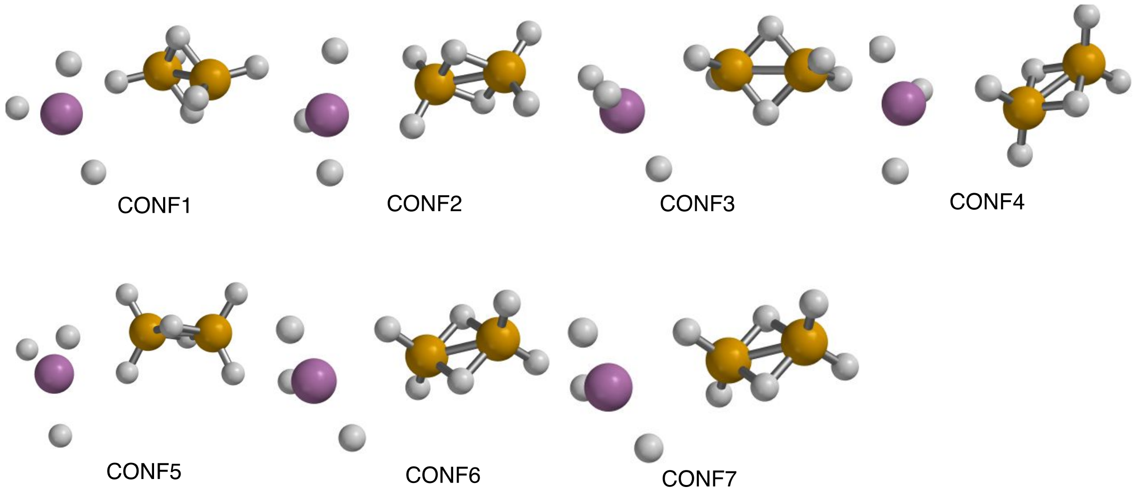

CONF#	*Energy(a.u.)*	*ΔGtot*
	wb97x-d3/def2-TZVPP	(kJ/mol)
CONF1	-297.4918694	3.9
CONF2	-297.4931053	0.2
CONF3	-297.4932029	**0**
CONF4	-297.4930328	0.25
CONF5	-297.4931894	0.05
CONF6	-297.4931181	0.2
CONF7	-297.493128	0.2

From the different conformers of the $(BCl_3)_2(AlCl_3)$ heterotrimers the 16 most stable were the following:

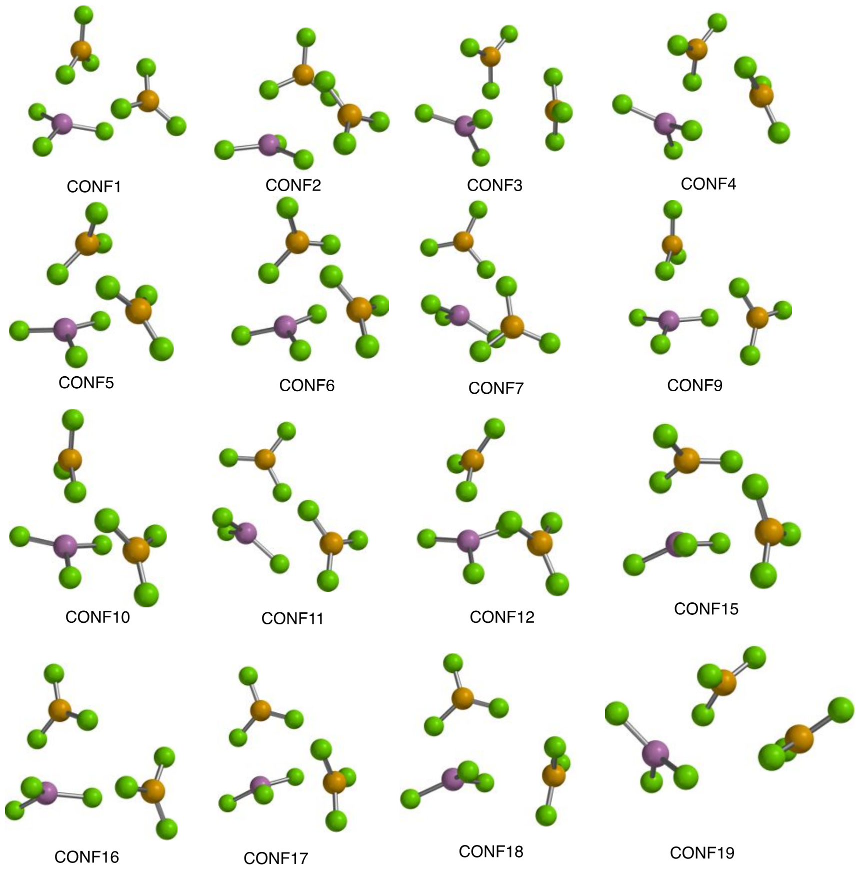

CONF#	*Energy (a.u.)* wb97x-d3/def2-TZVPP	*ΔGtot* (kJ/mol)
CONF1	-4434.527221	0
CONF2	-4434.526001	3.1
CONF3	-4434.526895	0.7
CONF4	-4434.526449	1.9
CONF5	-4434.52485	5.8
CONF6	-4434.524756	5.8
CONF7	-4434.525256	4.7
CONF9	-4434.525576	3.5
CONF10	-4434.525058	5.2
CONF11	-4434.525652	3.5
CONF12	-4434.525678	3.7
CONF15	-4434.525554	4.0
CONF16	-4434.525393	3.8
CONF17	-4434.525124	4.8
CONF18	-4434.524819	4.9
CONF19	-4434.525274	4.0

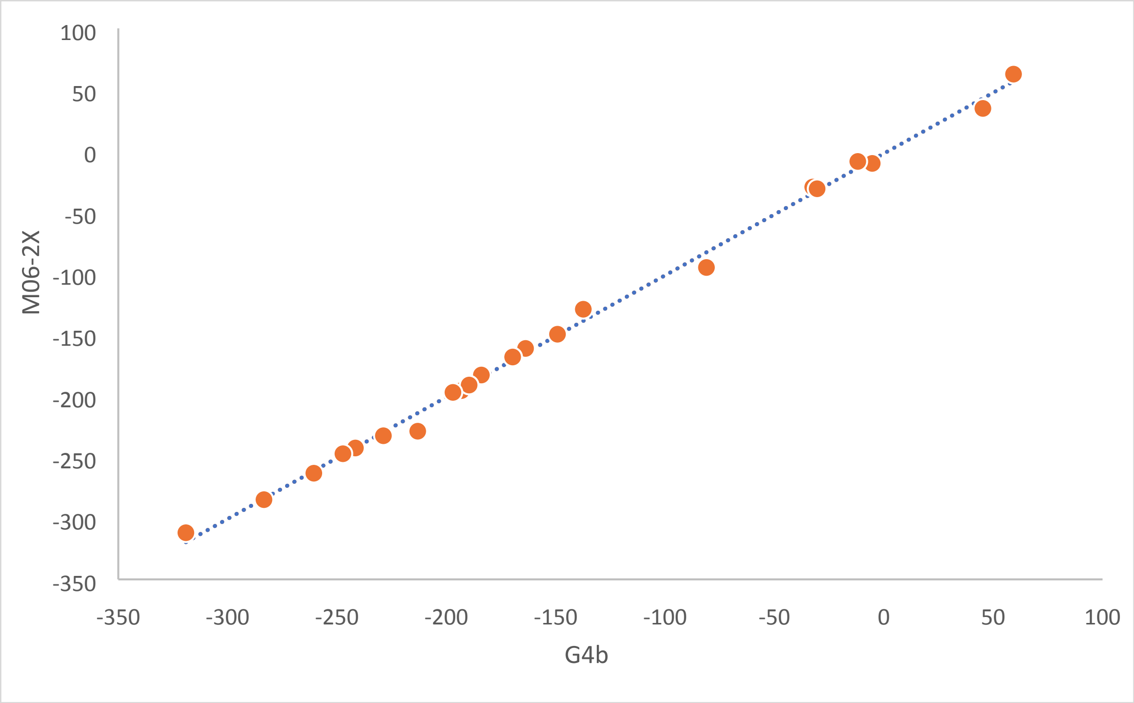

Figure S1. Linear correlation between the M06-2X and the G4b dimerization enthalpies for the BBX_6, $AlAlX_6$ and $BAlX_6$ (X = H, F, Cl) dimers and the B_3H_9, Al_3H_9, B_2AlH_9 and BAl_2H_9 trimers. All values in $kJ \cdot mol^{-1}$.

Table S2. LMO-EDA analysis for the BBX_6, $AlAlX_6$ and $BAlX_6$ (X = H, F, Cl) dimers (all values in kJ·mol^{-1})

a) Hydrides

Energies (kJ·mol^{-1}) of the different components				% of the attractive components to the total E		
Component	B_2H_6 (D_{2h})	Al_2H_6 (D_{2h})	$BAlH_6$ (C_{2v})	B_2H_6 (D_{2h})	Al_2H_6 (D_{2h})	$BAlH_6$ (C_{2v})
ELECTROSTATIC	-474.8	-423.1	-436.3	26.2	34.4	27.9
EXCHANGE	-560.2	-367.9	-485.1	30.9	29.9	31.0
REPULSION	1481.2	972.1	1254.2			
POLARIZATION	-659.3	-358.4	-544.9	36.4	29.1	34.8
**DISPERSION**	**-116.1**	**-82.0**	**-100.2**	**6.4**	**6.7**	**6.4**
TOTAL	-329.2	-259.3	-312.3			

b) Fluorides

Energies (kJ·mol^{-1}) of the different components					% of the attractive components to the total E			
Component	B_2F_6 (D_{2h})	B_2F_6 (C_{2h})	Al_2F_6 (D_{2h})	$BAlF_6$ (C_{2v})	B_2F_6 (D_{2h})	B_2F_6 (C_{2h})	Al_2F_6 (D_{2h})	$BAlF_6$ (C_{2v})
ELECTROSTATIC	-578.6	-20.1	-536.2	-558.7	31.7	26.0	43.6	36.1
EXCHANGE	-508.7	-14.2	-271.0	-399.0	27.9	18.3	22.0	25.8
REPULSION	1563.7	61.2	852.7	1225.9				
POLARIZATION	-599.6	-10.8	-314.6	-465.4	32.8	13.9	25.6	30.1
**DISPERSION**	**-139.2**	**-32.4**	**-109.2**	**-124.7**	**7.6**	**41.8**	**8.9**	**8.1**
TOTAL	-262.5	-16.4	-378.3	-321.9				

a) Chlorides

Energies (kJ·mol^{-1}) of the different components						% of the attractive components to the total E				
Component	B_2Cl_6 (D_{2h})	B_2Cl_6 (C_{2h})	Al_2Cl_6 (D_{2h})	$BAlCl_6$ (C_{2v})	$BAlCl_6$ (C_s)	B_2Cl_6 (D_{2h})	B_2Cl_6 (C_{2h})	Al_2Cl_6 (D_{2h})	$BAlCl_6$ (C_{2v})	$BAlCl_6$ (C_s)
ELECTROSTATIC	-458.5	-10.9	-374.6	-411.0	-74.6	23.8	14.5	30.5	26.2	22.6
EXCHANGE	-611.2	-15.1	-330.0	-470.5	-90.0	31.7	20.0	26.9	30.0	27.3
REPULSION	1753.1	61.7	950.0	1342.6	275.3					
POLARIZATION	-695.3	-5.4	-398.0	-544.3	-85.0	36.1	7.2	32.4	34.7	25.8
**DISPERSION**	**-161.8**	**-44.1**	**-124.0**	**-142.7**	**-79.8**	**8.4**	**58.3**	**10.1**	**9.1**	**24.2**
TOTAL	-173.6	-13.8	-276.6	-225.9	-54.1					

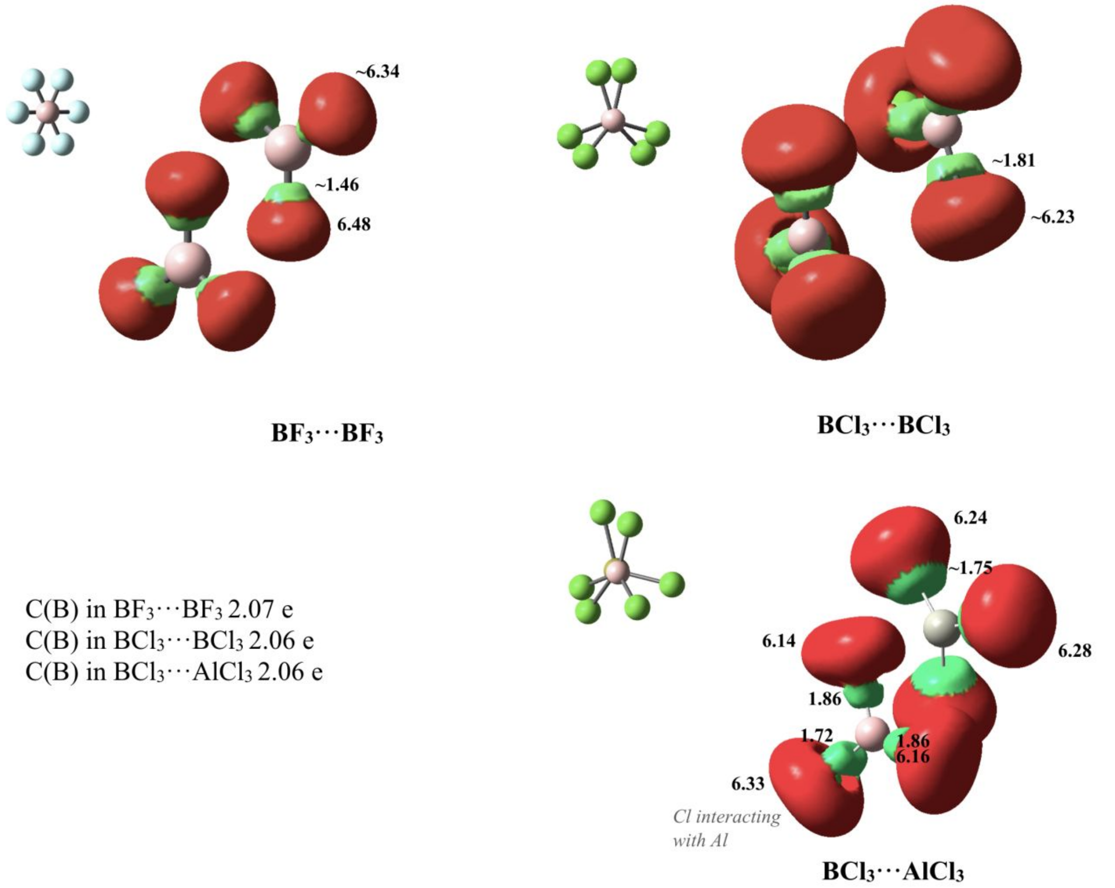

Figure S2. ELF basins of B_2F_6, B_2Cl_6 and $BAlCl_6$ dimers. ELF (0.8) disynaptic basins between a triel atom and a halogen atom appear in green, while lone pairs belonging to halogen atoms are colored in red. Populations are shown in atomic units (e).

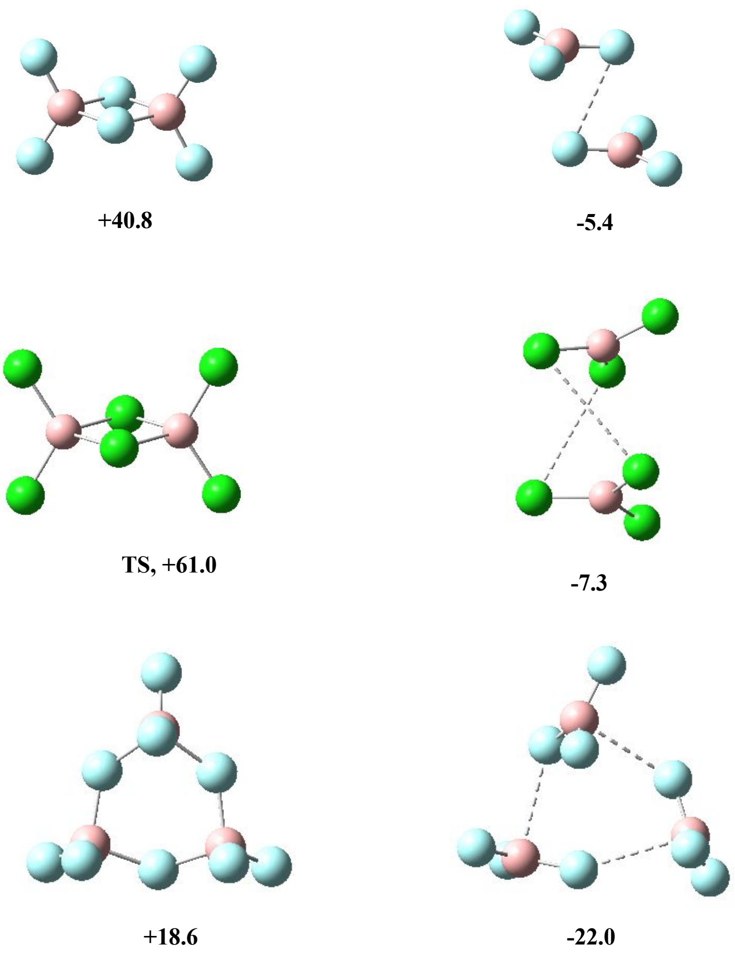

Figure S3. Structure and stabilization enthalpies (in kJ·mol^{-1}) of the BF_3 and BCl_3 dimers and the cyclic BF_3 trimers in water solution.

Table S3. AdNPD orbital list for diborane, dialane and H_3B-AlH_3 mixed dimer

BH_3-BH_3

---- AdNDP orbital list ----

#	1 Occ:	1.9929	Atom:	1B	2H		
#	2 Occ:	1.9929	Atom:	5B	7H		
#	3 Occ:	1.9929	Atom:	5B	6H		
#	4 Occ:	1.9929	Atom:	1B	3H		
#	5 Occ:	1.9984	Atom:	1B	5B	8H	
#	6 Occ:	1.9984	Atom:	1B	4H	5B	

Total occupation number in above orbitals: 11.97

Residual valence electrons of all atoms in the search list: 0.03

AlH_3-AlH_3

---- AdNDP orbital list ----

\# 1 Occ: 1.9966 Atom: 1Al 3H
\# 2 Occ: 1.9966 Atom: 5Al 7H
\# 3 Occ: 1.9966 Atom: 1Al 2H
\# 4 Occ: 1.9966 Atom: 5Al 8H
\# 5 Occ: 1.9787 Atom: 1Al 4H 5Al
\# 6 Occ: 1.9787 Atom: 1Al 5Al 6H
Total occupation number in above orbitals: 11.94
Residual valence electrons of all atoms in the search list: 0.06

BH_3-AlH_3

---- AdNDP orbital list ----

\# 1 Occ: 1.9971 Atom: 5Al 6H
\# 2 Occ: 1.9971 Atom: 5Al 8H
\# 3 Occ: 1.9923 Atom: 1B 3H
\# 4 Occ: 1.9923 Atom: 1B 2H
\# 5 Occ: 1.9903 Atom: 1B 5Al 7H
\# 6 Occ: 1.9903 Atom: 1B 4H 5Al
Total occupation number in above orbitals: 11.96
Residual valence electrons of all atoms in the search list: 0.04

BF_3-AlF_3

3c-2e model (the 18 lone pairs on the F atoms are not shown for simplicity)

---- AdNDP orbital list ----

\# 19 Occ: 1.9996 Atom: 1B 2F
\# 20 Occ: 1.9995 Atom: 1B 3F
\# 21 Occ: 1.9994 Atom: 5Al 6F
\# 22 Occ: 1.9994 Atom: 5Al 8F

\# 23 Occ: 1.9989 Atom: 1B 4F 5Al
\# 24 Occ: 1.9989 Atom: 1B 5Al 7F
Total occupation number in above orbitals: 47.17
Residual valence electrons of all atoms in the search list: 0.83

2c-2e model (the 16 lone pairs on the F atoms are not shown for simplicity)

\# 17 Occ: 1.9996 Atom: 1B 2F
\# 18 Occ: 1.9995 Atom: 1B 3F
\# 19 Occ: 1.9994 Atom: 5Al 6F

20 Occ: 1.9994 Atom: 5Al 8F
21 Occ: 1.9946 Atom: 1B 7F
22 Occ: 1.9946 Atom: 1B 4F
23 Occ: 1.9880 Atom: 4F 5Al
24 Occ: 1.9880 Atom: 5Al 7F

Total occupation number in above orbitals: 47.3836

Residual valence electrons of all atoms in the search list: 0.62

BCl_3-$AlCl_3$ (the 18 lone pairs on the Cl atoms are not shown for simplicity)

3c-2e model (the 18 lone pairs on the Cl atoms are not shown for simplicity)

---- AdNDP orbital list ----

19 Occ: 1.9990 Atom: 1Al 3Cl
20 Occ: 1.9990 Atom: 1Al 2Cl
21 Occ: 1.9985 Atom: 6B 7Cl
22 Occ: 1.9985 Atom: 6B 8Cl
23 Occ: 1.9929 Atom: 1Al 4Cl 6B
24 Occ: 1.9929 Atom: 1Al 5Cl 6B

Total occupation number in above orbitals: 46.61

Residual valence electrons of all atoms in the search list: 1.39

2c-2e model (the 16 lone pairs on the Cl atoms are not shown for simplicity)

17 Occ: 1.9990 Atom: 1Al 3Cl
18 Occ: 1.9990 Atom: 1Al 2Cl
19 Occ: 1.9985 Atom: 6B 7Cl
20 Occ: 1.9985 Atom: 6B 8Cl
21 Occ: 1.9904 Atom: 4Cl 6B
22 Occ: 1.9904 Atom: 5Cl 6B
23 Occ: 1.9844 Atom: 1Al 5Cl
24 Occ: 1.9844 Atom: 1Al 4Cl

Total occupation number in above orbitals: 47.11

Residual valence electrons of all atoms in the search list: 0.89

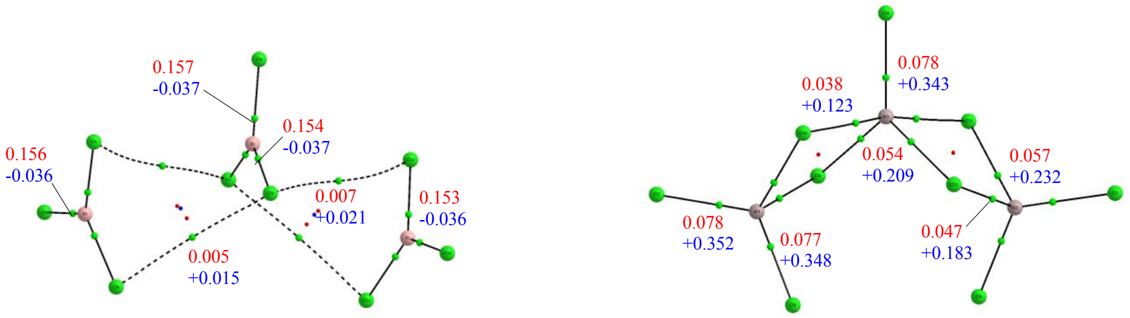

BCl_3-BCl_3-BCl_3 **$AlCl_3$-$AlCl_3$-$AlCl_3$**

Figure S4. Molecular graphs of linear B_3Cl_9 and Al_3Cl_9 clusters, showing the electron density (red) and its Laplacian (blue) (a.u.) at the corresponding BCPs.

Table S4. LMO-EDA analysis for the $BBBX_6$ and $AlAlAlX_6$ (X = H, F, Cl) homotrimers (all values in $kJ{\cdot}mol^{-1}$)

c) Hydrides

	Energies ($kJ{\cdot}mol^{-1}$) of the different components				% of the attractive components to the total E			
Component	$(BH_3)_3$ linear	$(BH_3)_3$ cyclic	$(AlH_3)_3$ linear	$(AlH_3)_3$ cyclic	$(BH_3)_3$ linear	$(BH_3)_3$ cyclic	$(AlH_3)_3$ linear	$(AlH_3)_3$ cyclic
ELECTROSTATIC	-733.6	-609.4	-659.8	-450.9	23.6	22.0	32.4	28.4
EXCHANGE	-1022.2	-857.5	-627.4	-446.6	32.9	31.0	30.8	28.2
REPULSION	2698.3	2188.7	1676.6	1149.6				
POLARIZATION	-1125.7	-1077.0	-586.6	-548.5	36.2	38.9	28.8	34.6
**DISPERSION**	**-224.5**	**-222.2**	**-162.1**	**-139.7**	**7.2**	**8.0**	**8.0**	**8.8**
TOTAL	-407.8	-577.4	-359.4	-436.2				

a) Fluorides

	Energies ($kJ{\cdot}mol^{-1}$) of the different components					% of the attractive components to the total E				
Component	$(BF_3)_3$ linear	$(BF_3)_3$ Cyclic C_{3v}	$(BF_3)_3$ Cyclic C_1	$(AlF_3)_3$ linear	$(AlF_3)_3$ Cyclic C_{3v}	$(BF_3)_3$ linear	$(BF_3)_3$ Cyclic C_{3v}	$(BF_3)_3$ Cyclic C_1	$(AlF_3)_3$ linear	$(AlF_3)_3$ Cyclic C_{3v}
ELECTROSTATIC	-38.4	-766.9	-49.5	-929.5	-685.0	25.6	29.2	26.4	40.7	39.9
EXCHANGE	-27.2	-688.6	-32.1	-531.9	-315.9	18.1	26.2	17.1	23.3	18.4
REPULSION	118.6	2140.4	143.6	1669.3	1043.2					
POLARIZATION	-20.8	-931.0	-27.8	-595.1	-543.8	13.8	35.4	14.8	26.0	31.7
**DISPERSION**	**-63.6**	**-241.2**	**-78.2**	**-229.5**	**-170.2**	**42.4**	**9.2**	**41.7**	**10.0**	**9.9**
TOTAL	-31.4	-487.3	-43.9	-616.8	-671.7					

b) Chlorides

	Energies ($kJ{\cdot}mol^{-1}$) of the different components					% of the attractive components to the total E				
Component	$(BCl_3)_3$ linear	$(BCl_3)_3$ Cyclic C_{3v}	$(BCl_3)_3$ Cyclic C_1	$(AlCl_3)_3$ linear	$(AlCl_3)_3$ Cyclic C_{3v}	$(BCl_3)_3$ linear	$(BCl_3)_3$ cyclic	$(BCl_3)_3$ cyclic	$(AlCl_3)_3$ linear	$(AlCl_3)_3$ Cyclic C_{3v}
ELECTROSTATIC	-21.8	-665.2	-26.5	-587.9	-486.1	14.4	22.8	15.5	28.1	27.0
EXCHANGE	-30.4	-902.0	-35.6	-603.2	-452.8	20.1	31.0	20.9	28.8	25.2
REPULSION	123.8	2579.1	141.2	1730.0	1331.8					
POLARIZATION	-10.9	-1043.9	-11.8	-634.1	-622.0	7.2	35.8	6.9	30.3	34.6
**DISPERSION**	**-88.3**	**-301.3**	**-96.9**	**-269.2**	**-238.4**	**58.3**	**10.3**	**56.7**	**12.9**	**13.3**
TOTAL	-27.6	-333.3	-29.7	-364.3	-467.5					

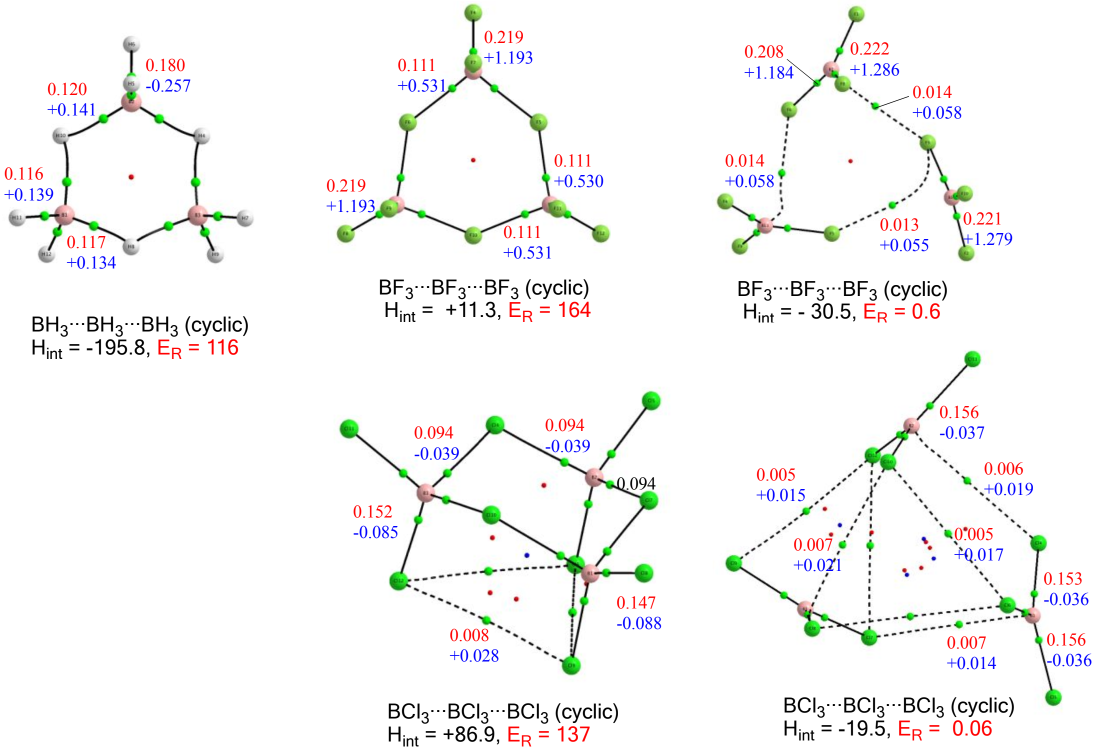

Figure S5. Molecular graphs of global different cyclic isomers of B_3X_9 (X = H, F, Cl) ternary complexes, showing electron density (red) and its Laplacian (blue) (a.u.) at the corresponding BCPs. The interaction enthalpies (H_{int}) and the monomer distortion energy (E_R) (in red) are provided. All values in kJ·mol^{-1}.

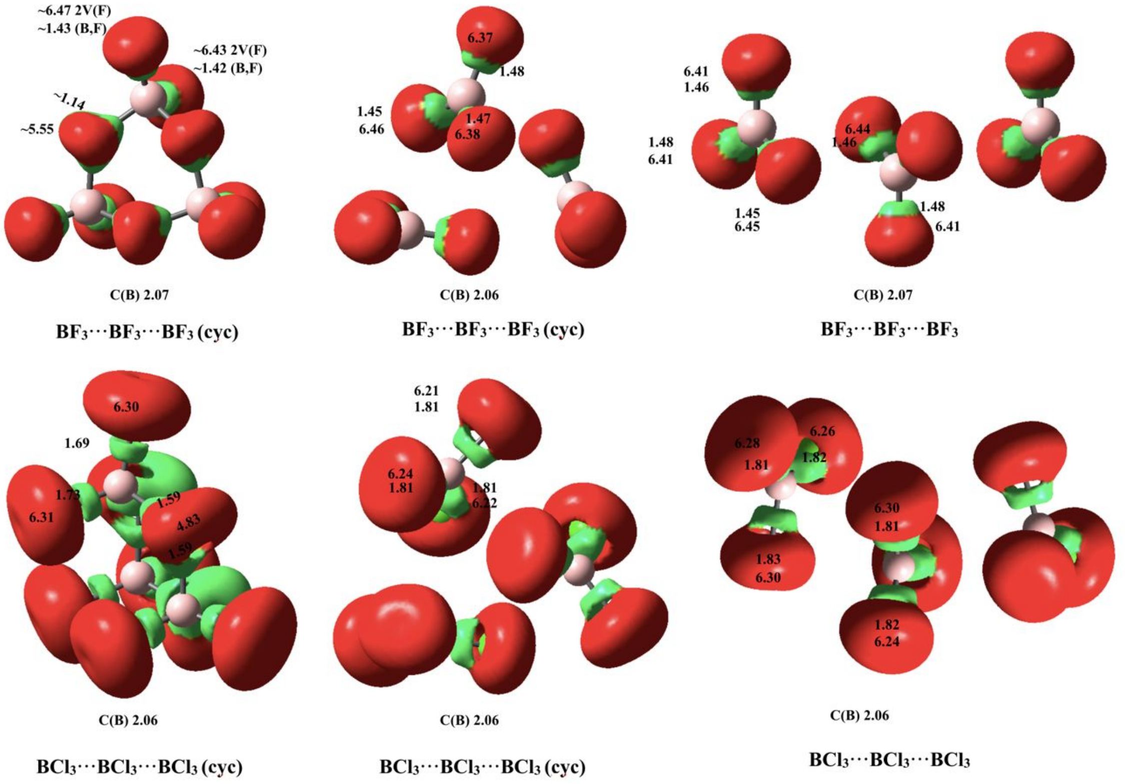

Figure S6. ELF basins of linear and cyclic B_3F_9 and B_3Cl_9 trimers. ELF (0.8) disynaptic basins between a triel atom and a halogen atom appear in green, while lone pairs belonging to halogen atoms are colored in red. Populations are shown in atomic units (e).

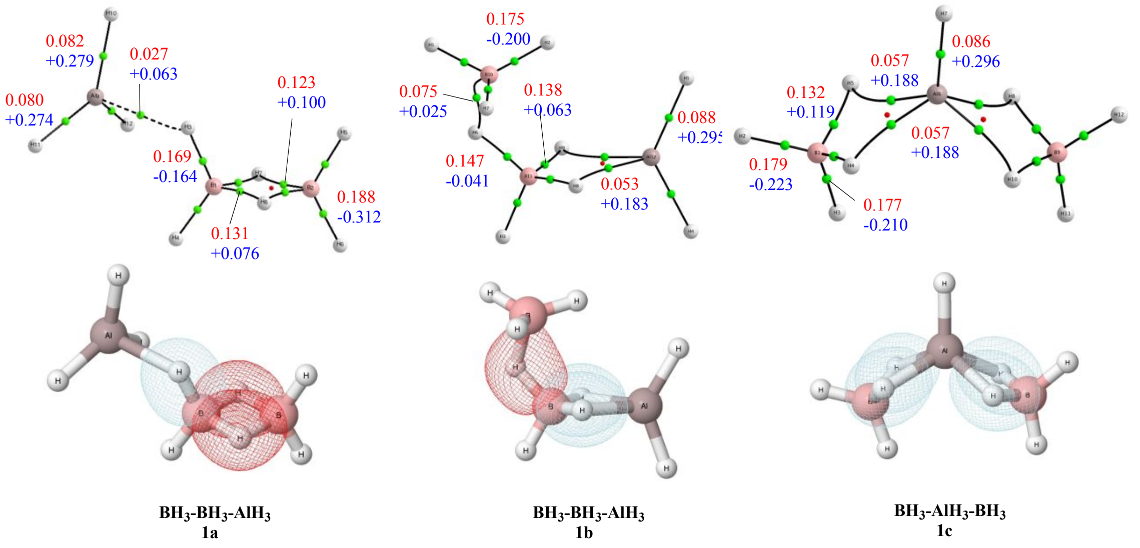

Figure S7. Fist row, molecular graphs for trimers **1a**,**1b** and **1c** of Figure 7. Second row, AdNDP 3c-2e MOs for the same systems, showing the electron density (red) and its

Laplacian (blue) (a.u.) at the corresponding BCPs. Red and blue colors are used for MOs involving exclusively B and for MOs involving Al, respectively.

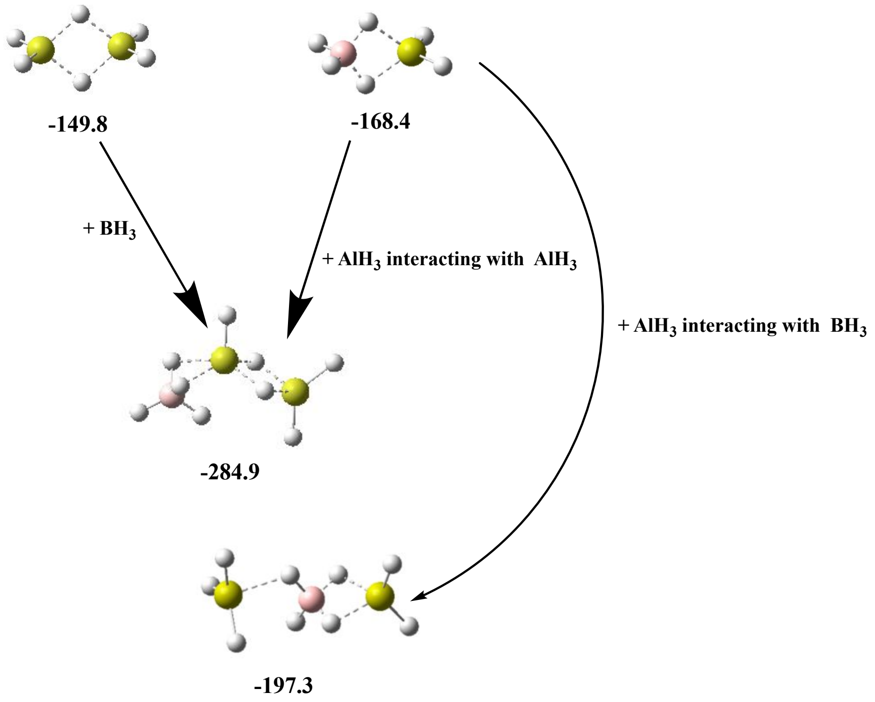

Figure S8. Schematic representation of the possible mechanisms associated with the formation of BH_3-AlH_3-AlH_3 isomers. The stabilization enthalpies are in kJ·mol^{-1}.

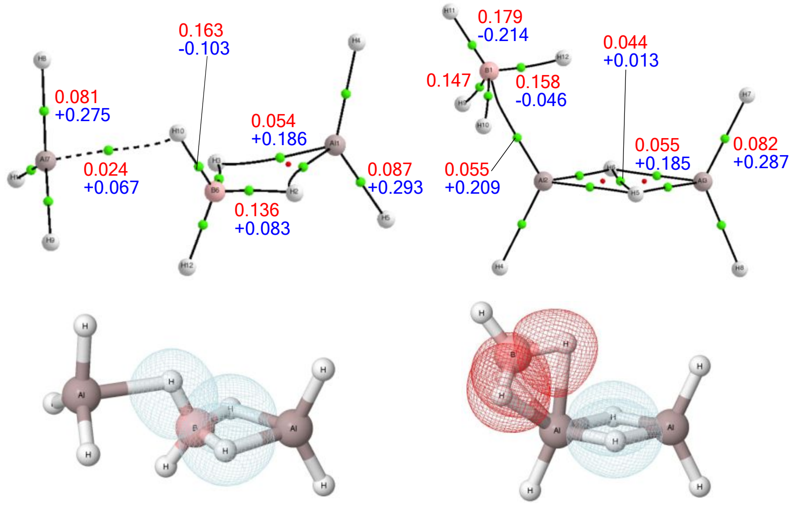

AlH_3-BH_3-AlH_3 BH_3-AlH_3-AlH_3

Figure S9. Fist row, molecular graphs for trimers AlH_3-BH_3-AlH_3 and of BH_3-AlH_3-AlH_3. Second row, AdNDP 3c-2e MOs for the same systems, showing the electron density (red) and its Laplacian (blue) (a.u.) at the corresponding BCPs. Red and blue colors are used for MOs involving exclusively B and for MOs involving Al, respectively.

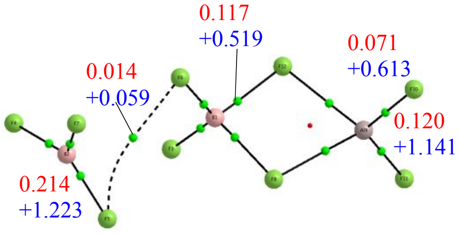

$BF_3\cdots BF_3\cdots AlF_3$

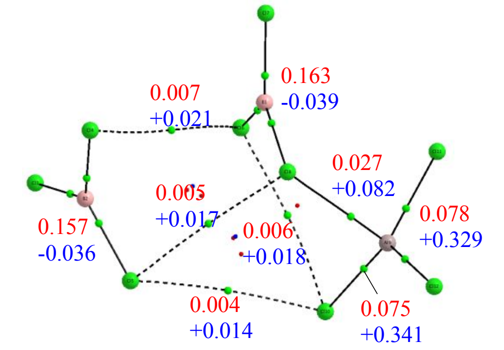

$BCl_3\cdots BCl_3\cdots AlCl_3$

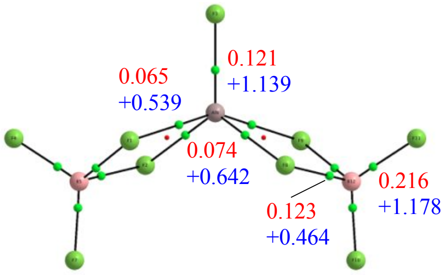

$BF_3\cdots AlF_3\cdots BF_3$

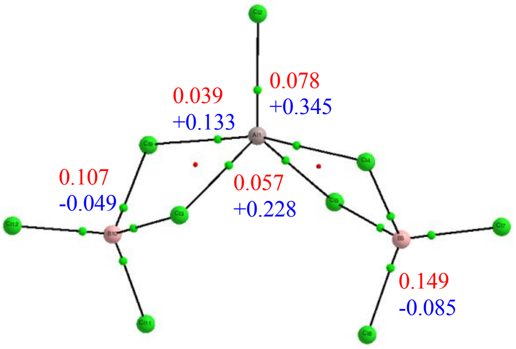

$BCl_3\cdots AlCl_3\cdots BCl_3$

Figure S10. Molecular graphs of B_2AlF_9 and B_2AlCl_9 ternary complexes, showing the electron density (red) and its Laplacian (blue) (a.u.) at the corresponding BCPs.

Table S5. LMO-EDA analysis for the $BBAlX_6$ and $BAlAlX_6$ (X = H, F, Cl) heterotrimers (all values in $kJ \cdot mol^{-1}$)

a) Hydrides

	Energies ($kJ \cdot mol^{-1}$) of the different components						% of the attractive components to the total E					
Component	$BBAlH_9$ linear	$BBAlH_9$ cyclic	$BAlBH_9$ linear	$BAlAlH_9$ linear	$AlBAlH_9$ cyclic	$AlBAlH_9$ linear	$BBAlH_9$ linear	$BBAlH_9$ cyclic	$BAlBH_9$ linear	$BAlAlH_9$ linear	$AlBAlH_9$ cyclic	$AlBAlH_9$ linear
ELECTROSTATIC	-535.0	-596.2	-802.8	-1027.8	-605.2	-471.9	33.7	37.6	50.6	64.8	38.2	29.8
EXCHANGE	-691.7	-800.4	-950.9	-1114.8	-733.2	-549.9	43.6	50.5	60.0	70.3	46.2	34.7
REPULSION	1767.4	2007.2	2479.9	2917.3	1829.3	1425.3						
POLARIZATION	-743.6	-953.1	-1078.6	-1208.5	-838.6	-615.5	46.9	60.1	68.0	76.2	52.9	38.8
**DISPERSION**	**-177.2**	**-206.2**	**-206.2**	**-205.1**	**-187.2**	**-145.6**	**11.2**	**13.0**	**13.0**	**12.9**	**11.8**	**9.2**
TOTAL	-379.9	-548.7	-558.6	-639.1	-534.7	-357.6						

b) Fluorides

	Energies ($kJ \cdot mol^{-1}$) of the different components						% of the attractive components to the total E					
Component	$BBAlF_9$ linear	$BBAlF_9$ cyclic	$BAlBF_9$ linear	$BAlAlF_9$ linear	$AlBAlF_9$ cyclic	$AlBAlH_9$ linear	$BBAlF_9$ linear	$BBAlF_9$ cyclic	$BAlBF_9$ linear	$BAlAlF_9$ linear	$AlBAlF_9$ cyclic	$AlBAlF_9$ linear
ELECTROSTATIC	-571.5	-749.0	-959.5	-942.9	-697.8	-647.5	35.2	31.7	34.1	37.1	34.8	33.9
EXCHANGE	-413.8	-578.1	-749.1	-638.9	-439.4	-484.2	25.5	24.5	26.7	25.1	21.9	25.3
REPULSION	1290.3	1809.5	2316.1	1988.5	1400.8	1510.7						
POLARIZATION	-482.6	-813.8	-846.0	-718.9	-680.2	-592.7	29.7	34.5	30.1	28.3	33.9	31.0
**DISPERSION**	**-156.8**	**-220.4**	**-255.2**	**-242.1**	**-188.2**	**-186.9**	**9.7**	**9.3**	**9.1**	**9.5**	**9.4**	**9.8**
TOTAL	-334.5	-551.7	-493.8	-554.2	-604.8	-400.6						

c) Chlorides

	Energies ($kJ \cdot mol^{-1}$) of the different components						% of the attractive components to the total E					
Component	$BBAlCl_9$ linear	$BBAlCl_9$ cyclic	$BAlBCl_9$ linear	$BAlAlCl_9$ linear	$AlBAlCl_9$ cyclic	$AlBAlCl_9$ linear	$BBAlCl_9$ linear	$BBAlCl_9$ cyclic	$BAlBCl_9$ linear	$BAlAlCl_9$ linear	$AlBAlF_9$ cyclic	$AlBAlCl_9$ linear
ELECTROSTATIC	-87.0	-141.5	-151.4	-388.4	-538.9	-512.1	20.8	31.4	25.0	29.2	25.2	25.4
EXCHANGE	-108.4	-176.9	-191.7	-355.8	-595.7	-597.2	25.9	39.3	31.7	26.8	27.8	29.6
REPULSION	347.8	508.8	546.8	1039.9	1724.5	1719.3						
POLARIZATION	-92.6	-213.3	-193.1	-404.2	-750.2	-677.7	22.2	47.4	31.9	30.4	35.0	33.6
**DISPERSION**	**-129.9**	**-66.0**	**-68.5**	**-179.7**	**-258.0**	**-230.9**	**31.1**	**14.7**	**11.3**	**13.5**	**12.0**	**11.4**
TOTAL	-70.2	-89.0	-57.9	-288.2	-418.4	-298.6						

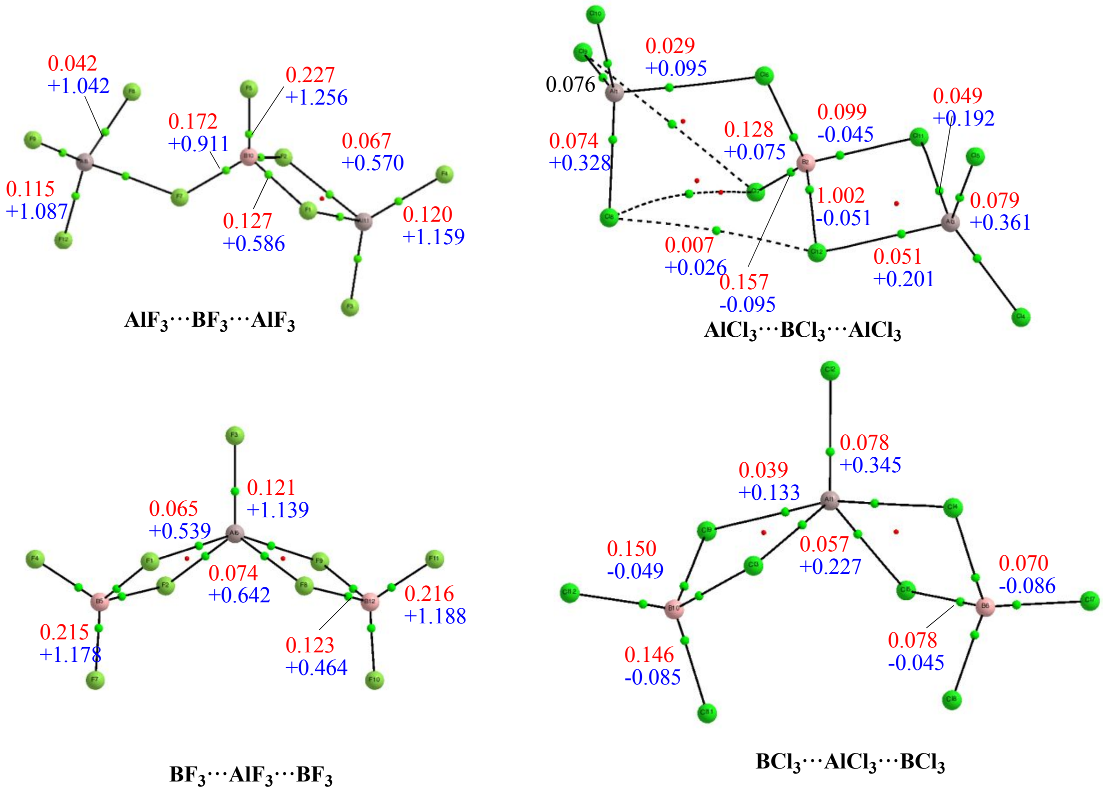

Figure S11. Molecular graphs of $AlX_3BX_3AlX_3$ (X = F, Cl) and $BX_3AlX_3BX_3$ (X = F, Cl) ternary complexes, showing the electron density (red) and its Laplacian (blue) (a.u.) at the corresponding BCPs.

Table S6. MBIE analysis for the cyclic mixed B_2AlF_9, BAl_2F_9, B_2AlCl_9 and BAl_2Cl_9 ternary complexes. All values in kJ·mol^{-1}.

Ternary complex	$E_R(A)$	$E_R(B)$	$E_R(C)$	$\Delta^2(AB)$	$\Delta^2(AC)$	$\Delta^2(BC)$	$\Delta^3(ABC)$	E_{total}
B_2AlF_9 (Cyclic)	176.6	155.9	77.9	-101.3	-154.8	-151.7	-143.4	-140.8
BAl_2F_9(Cyclic)	74.7	80.0	164.4	-192.1	-139.5	-139.4	-133.4	-285.5
B_2AlCl_9 (Cyclic)	138.9	138.6	78.7	-63.4	-79.6	-102.2	-124.1	-13.1
BAl_2Cl_9 (Cyclic)	75.9	140.0	81.1	-83.4	-128.3	-100.9	-101.7	-117.3
